# A computational method for prioritizing targeted therapies in precision oncology: performance analysis in the SHIVA01 trial

**DOI:** 10.1038/s41698-021-00191-2

**Published:** 2021-06-23

**Authors:** Istvan Petak, Maud Kamal, Anna Dirner, Ivan Bieche, Robert Doczi, Odette Mariani, Peter Filotas, Anne Salomon, Barbara Vodicska, Vincent Servois, Edit Varkondi, David Gentien, Dora Tihanyi, Patricia Tresca, Dora Lakatos, Nicolas Servant, Julia Deri, Pauline du Rusquec, Csilla Hegedus, Diana Bello Roufai, Richard Schwab, Celia Dupain, Istvan T. Valyi-Nagy, Christophe Le Tourneau

**Affiliations:** 1 Department of Pharmacology and Pharmacotherapy, Semmelweis University, Budapest, Hungary; 2 Department of Biopharmaceutical Sciences, University of Illinois at Chicago, Chicago, USA; 3 Oncompass Medicine, Budapest, Hungary; 4 Department of Drug Development and Innovation (D3i), Institute Curie, Paris & Saint-Cloud, France; 5 Pharmacogenomics unit, Institut Curie, Paris, France; 6 Department of Biopathology, Institut Curie, Paris, France; 7 Department of Radiology, Institut Curie, Paris, France; 8 Translational Research Department, Institut Curie, Paris, France; 9 INSERM U900 Research Unit, Paris & Saint-Cloud, France; 10 Central Hospital of Southern Pest—National Institute for Hematology and Infectious Diseases, Budapest, Hungary; 11 Paris-Saclay University, Paris, France

**Keywords:** Cancer therapy, Cancer models

## Abstract

Precision oncology is currently based on pairing molecularly targeted agents (MTA) to predefined single driver genes or biomarkers. Each tumor harbors a combination of a large number of potential genetic alterations of multiple driver genes in a complex system that limits the potential of this approach. We have developed an artificial intelligence (AI)-assisted computational method, the digital drug-assignment (DDA) system, to prioritize potential MTAs for each cancer patient based on the complex individual molecular profile of their tumor. We analyzed the clinical benefit of the DDA system on the molecular and clinical outcome data of patients treated in the SHIVA01 precision oncology clinical trial with MTAs matched to individual genetic alterations or biomarkers of their tumor. We found that the DDA score assigned to MTAs was significantly higher in patients experiencing disease control than in patients with progressive disease (1523 versus 580, *P* = 0.037). The median PFS was also significantly longer in patients receiving MTAs with high (1000+ <) than with low (<0) DDA scores (3.95 versus 1.95 months, *P* = 0.044). Our results indicate that AI-based systems, like DDA, are promising new tools for oncologists to improve the clinical benefit of precision oncology.

## Introduction

Precision oncology, the molecularly targeted treatment of every cancer patient based on the individual genetic alterations of their tumor, is a highly anticipated, straightforward approach to beat cancer eventually. Efforts in precision oncology have been focusing on identifying single variables, predictive biomarkers, presence or absence of molecular alterations, able to predict alone the response to a molecularly targeted agent (MTA). This approach has led to the development of predictive companion diagnostic tests that are now part of the indication of many MTAs^1,2^. The introduction of predictive biomarkers in the drug discovery and clinical use of MTAs was a significant milestone in precision oncology, but using single biomarkers has severe limitations.

Basket trials to find successful predictive biomarker–MTA pairs are in progress. The NCI-MATCH trial is a very important example. In NCI-MATCH tumors are molecularly profiled, and patients are assigned to different treatment arms based on the presence of predefined single biomarkers. The trial is feasible, and in some cases, successful, but it also indicates the limitations of the single biomarker paradigm as only three of the first 11 completed subprotocols resulted in positive results^3^. TAPUR, the other large-scale precision oncology basket trial organized by ASCO (American Society of Clinical Oncology), has reported similar results^4^.

Other clinical trials have been designed to analyze the overall clinical benefit of treating patients based on their tumors’ molecular profile with matching “on-target” but off-label MTAs or with matching investigational MTAs. IMPACT01 was the longest-running precision oncology trial that evaluated the benefit and limitation of matching MTAs to single biomarkers^5^. The ten-year survival rate was 6% in case molecularly matching therapies versus 1% in non-matching therapies (*P* < 0.0001). The objective response rate and the clinical benefit rate (including stable disease) were higher in case of matched MTAs compared to the results of the non-matched treatments (16.4% versus 5.4% and 35% versus 20.3%, respectively)^5^. The other pioneering precision oncology trial, MOSCATO01, found an 11% response rate and 33% benefit based on >1.3 longer PFS2/PFS1 ratio^6^. These results indicate that matching MTAs to single biomarkers does provide benefit; however, SHIVA01, the first randomized precision oncology trial assessing the efficacy of matching MTAs compared to conventional chemotherapy, did not find any statistically significant advantage of this approach^7^.

ESCAT, the European Society for Medical Oncology (ESMO) Scale for Clinical Actionability of molecular Targets, defines tiers to prioritize MTAs based on the highest level of evidence linking the MTA to a genetic alteration or a biomarker present in the patient’s tumor^8^. Tier I includes molecular alterations, which have demonstrated sufficient and robust clinical evidence to predict response to an MTA. Tier II includes molecular alterations demonstrating less robust clinical evidence in the same tumor type. Tier III includes alterations with evidence generated in other tumor types. Within tier III, tier IIIB alterations are different genetic variants of the same gene. Tier IV alterations are supported by preclinical data or in silico models only.

We have retrospectively evaluated the efficacy of matched MTA given in SHIVA01, also according to ESCAT tiers^9^. Molecular alterations revealed in SHIVA01 were retrospectively classified into the ESCAT tiers, then the average PFS and OS of MTAs in different tiers were compared using log-rank tests. We did not find a significant difference in the clinical outcome of the MTAs in tiers II, IIIA, and IV. Tier IIIB MTAs were associated with significantly shorter PFS than the MTAs in the other ESCAT tiers. This result indicates that the correct functional classification of the molecular alteration was a critical factor of success, but the level of evidence that links the MTA to the molecular alteration was not a significant predictor of response to the MTAs in the SHIVA01 trial. COSMIC (Catalogue of Somatic Mutations in Cancer) contains more than six million different potential somatic genetic alterations in more than 600 cancer genes^10^. The functional significance of most of these alterations is unknown (variants of unknown significance, VUS) or is associated with conflicting or limited evidence in the literature. This is a significant obstacle that contributes to the limited success rate of precision oncology today.

Another critical factor in precision oncology is that each tumor harbors a combination of 3–4 genetic driver alterations on average, and additional driver alterations in the non-coding regions are also present, raising the average number of molecular driver alterations to an average of 4–5 in every tumor^11^.

The I-PREDICT study presented evidence that MTA treatments (mostly combinations) inhibiting more than half (>50%) of the driver genetic alterations present in the tumor (high “matching score”) achieved at least 30% longer PFS (PFS2/PFS1 ≥ 1.3) compared to the previous line of standard therapy in 75% of patients, in comparison to 36.6% success rate of patients treated with MTAs targeting less than 50% of drivers (low “matching score”)^12^. This result indicates that the efficacy of MTAs matching their biomarker or driver is limited by the presence of unmatched drivers in two-thirds of patients.

Due to the recent outstanding advancement of molecular diagnostics, especially next-generation sequencing (NGS), it is now possible to analyze multiple driver genes in parallel instead of one by one, even in routine clinical practice. The challenge of precision oncology today is to assess the functional significance of all detected genetic alterations of all potential driver genes (single-nucleotide variations (SNVs), indels, CNVs, structural changes), next choosing the right target and the matching MTA that can be effective in the presence of the unmatched drivers. The complexity is further increased by the different sensitivity of individual genetic alterations to different MTAs matching the same target.

A potential solution is the deployment of standardized computational methods, artificial intelligence (AI)-assisted molecular drug-assignment algorithms. Computational tools can handle multi-dimensional, multianalyte data, and provide reproducible results. Therefore, computational algorithms are feasible tools to forecast treatment response to different MTAs in the case of the multiple different combinations present in tumors, and their performance can be evaluated as medical devices.

In this study, we aimed to develop a computational method (“digital drug assignment,” DDA), to test its performance to identify the most likely effective targeted therapies for cancer patients based on the individual complex molecular profile of their tumor. Driver genes and their specific genetic alterations are not equally important in the survival of the cancer cell. Several driver genes can be associated with multiple MTAs, and many MTAs can be associated with multiple drivers directly or indirectly through associated indirect molecular targets in the same tumor. The presence and importance of the associations between the drivers, targets, and MTAs are supported by variable numbers and level of experimental evidence. The goal was to create a system that automatically prioritizes potential driver genes and associated druggable targets based on thousands of available functional interactions derived from published evidence. Next, identifies MTAs that inhibit the most important drivers or the most important druggable targets associated with the most important divers at the highest level of aggregated evidence.

In theory, a system like this could maximize the chance to identify the MTA, which most likely triggers a therapeutic response based on our current knowledge of cancer biology and molecular pharmacology.

The DDA system presented in this study consists of a curated evidence-based network of over 12,000 driver-target-MTA interactions implemented in a software system, the Realtime Oncology Treatment Calculator^TM^. The DDA system automatically assigns a mathematical DDA score, the “aggregated evidence level” (AEL), to MTAs related to cancer patients’ complete molecular profile in milliseconds.

Next, we used the molecular profile and the clinical outcome data of patients treated in the SHIVA01 clinical trial to evaluate the clinical performance of DDA. In SHIVA01, all patients in the precision oncology arm were treated with an MTA matched to a predefined biomarker present in the patient’s tumor. However, we found that MTAs with higher DDA scores were associated with significantly higher clinical benefit.

Results presented here indicate that DDA can potentially overcome the limitations of single biomarker-based treatment decisions, address the molecular complexity of cancer to improve the clinical benefit of precision oncology.

## Results

### Overview of the DDA

Today, AI often refers to only machine learning (ML). However, computer science and the US Food and Drug Administration (FDA) action plan consider AI any intelligent computer programs that improve diagnostics or treatment decisions^13,14^. It would require vast clinical databases to develop effective predictive algorithms for treatment decisions solely with ML due to the large number of parameters that can influence the response to MTAs. Therefore, we decided to use a complex (“if–then”) rule-based expert system to create the DDA system based on the functional knowledge (“what we already know”) available from published experimental data. Rule-based expert systems are transparent, open box systems, and provide consistent results. Later, parts of the system can be further improved with ML methods after the fractionization of current complexity.

**Fig. 1:**
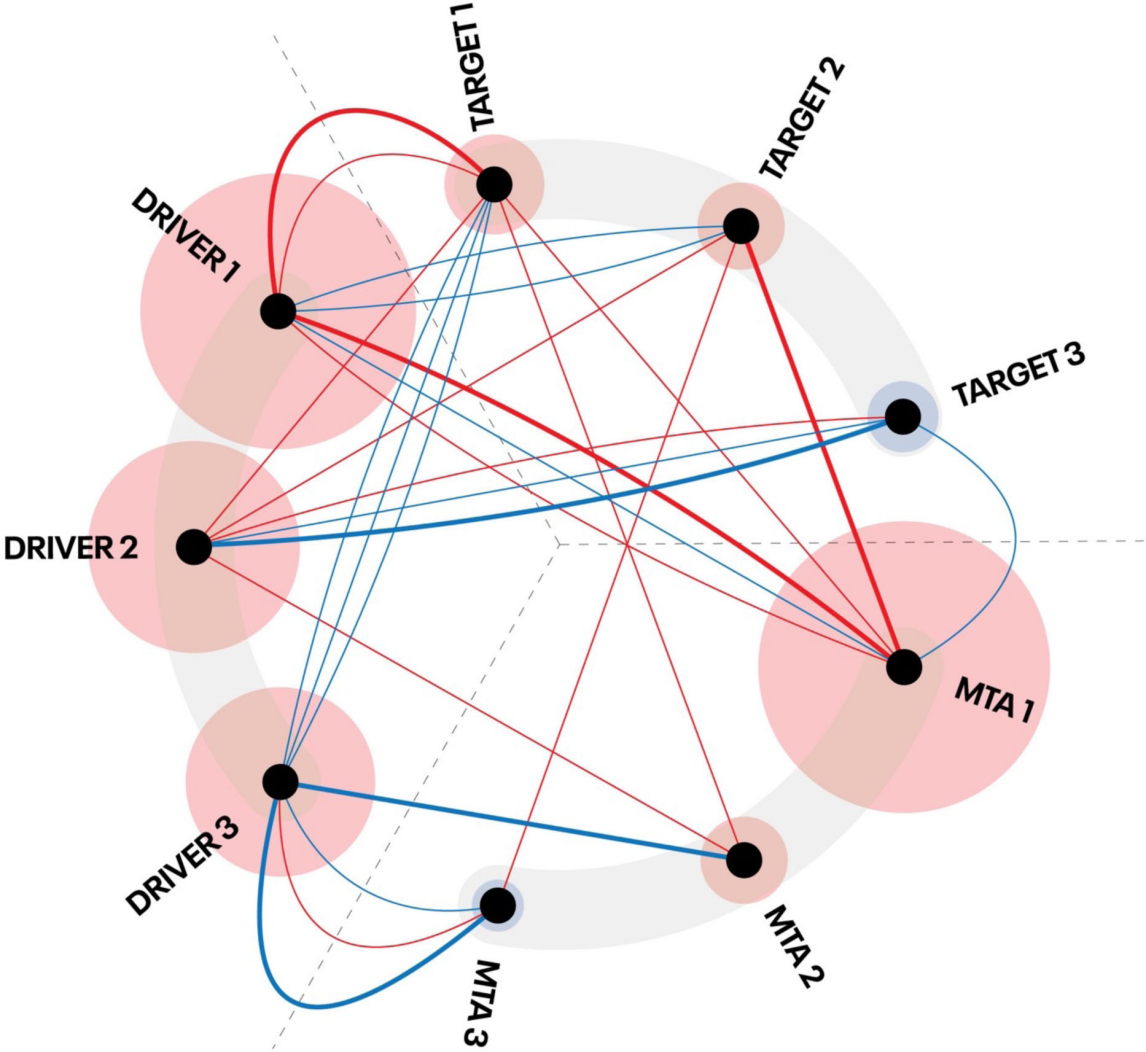
Schematic representation of the system and method of digital drug assignment (DDA). A network of published evidence-based functional associations between potential driver cancer genes (DRIVER) with genetic alterations, druggable molecular targets (TARGET), and molecularly targeted agents (MTA) is created. A mathematical DDA score (“aggregated evidence level”, AEL) of each driver gene present in the tumor and their associated targets and MTAs is calculated by aggregating the number and weight of positive (red lines) and negative (blue lines) associations. The interactions represented by the lines are, therefore, not physical associations but evidence-based functional associations and count both ways in the calculation of the AEL score of DRIVERS, TARGETS, and MTAs. The thickness of the lines represents the weight (evidence level) of an individual association. The final AEL of an MTA is determined by the number and weight of direct associations with drivers present in the tumor, the number and weight of associations between the drivers and the targets of the MTA, and the AEL of the drivers and targets associated with the MTA. The size of the outer circle around the dots represents the AEL of DRIVERS, TARGETS, and MTAs. The red color of the outer circle indicates positive, and the blue color of the outer circle indicates a negative AEL score based on the aggregated AEL of the AEL of all associations. In the case of the DRIVERS, all positive and negative associations support the functional significance of a DRIVER. Therefore, the AEL is still positive if the number and weight of positive associations outweigh the number and weight of negative associations. The system is designed to identify the MTA associated with the most important druggable TARGETS associated with the most important DRIVERS. This drawing is simplified for better clarity. For a typical tumor, the number of evidence-based interactions links three DRIVERS to 50–300 MTAs of 5–20 TARGETS by 500–1500 evidence-based interactions.

DDA uses a network of functional associations among mutant driver genes harboring molecular alterations, including SNVs, CNVs, and gene expression, tumor mutation burden (TMB), microsatellite instability (MSI) and druggable targets, MTAs, and tumor type (localization and histology) based on published peer-reviewed evidence (PubMed) built into a software system, the Realtime Oncology Treatment Calculator^TM^ (Fig. 1). Information about the frequency of molecular alterations was also included (COSMIC)^10^.

DDA algorithm assigns a score to each gene harboring molecular alterations (“driver calculation”) based on the number and weight of all functional associations with druggable target genes and MTAs. Next, DDA assigns a score to druggable target genes (“target calculation”) based on the number and weight of associations with all potential driver genes in the tumor. Finally, scores of MTAs are calculated based on the number and weight of associations with all “driver” genes and all “target” genes and the score of these genes. The weight of any association is calculated using constants (“matching weights”) corresponding to the similarity between the biological parameters of the particular tumor and the same parameters in the evidence used to generate the association.

### “Driver calculation”, prioritization of the driver genes by the DDA system

First, a mathematical DDA score, the AEL of each mutant gene present in a tumor, is calculated based on published evidence of the functional significance of the particular mutation and evidence of other mutations found in other patients in the same gene or the gene as a driver gene in general in the evidence database (“driver calculation”). Functional evidence is a piece of evidence that indicates the role of the gene in the carcinogenesis in the patient’s tumor type or another tumor type, or evidence for the functional significance of the mutant gene and molecular alterations in the gene in the sensitivity or resistance (preclinical or clinical) to MTAs directly targeting the gene or an indirect druggable target gene that is functionally linked to the mutant gene. Each association that supports the functional relevance of the mutant gene, molecular all potential alterations in the mutant gene, the particular molecular alteration present in the patient’s tumor, counts as a positive association and each association indicating the functional irrelevance of the mutant gene or the molecular alterations in the mutant gene, the particular molecular alteration present in the patient’s tumor, counts as a negative association.

The evidence level (weight) of the associations is calculated based on the type (clinical or preclinical), source of the evidence (journal), and relevance (matching tumor type and matching molecular alteration type/exact alteration) in the particular patient. Whole available supporting evidence is aggregated to calculate the AEL of each specific mutation and all mutant genes in the same patient. All mutant genes with an AEL score higher than zero are considered a potential driver in further calculations.

### “Target calculation”, prioritization of the druggable targets by the DDA system

Next, the AEL of druggable genes (drivers or indirect targets) is calculated based on all available evidence which links each druggable gene to all potential driver genes present in the same patient (“target calculation”). Associations between the potential driver genes and a target gene, which indicate that drugs targeting the druggable target gene are more effective in the presence of a driver gene count as a positive association, associations which indicate that drugs targeting the target gene in the presence of the driver is less effective counts as a negative association. The evidence level of all druggable targets’ associations with all mutant genes in the same patient present is aggregated to calculate the AEL score of the druggable target.

### “MTA calculation”: prioritization of the MTAs by the DDA system

Finally, the AEL score of potential MTAs is calculated based on the AEL of all associations between the MTA and all potential drivers and associated druggable targets (“MTA calculation”). Associations between the potential driver genes and an MTA indicating that the MTA is more effective in the presence of the potential driver gene, or the particular molecular alteration of the driver gene, are calculated as positive associations. Associations between the potential driver genes and an MTA indicating that the MTA is less effective in the presence of the potential driver gene or the particular molecular alteration of the driver gene are calculated as negative associations. The AEL scores of the targets with a positive aggregated score are added to the AEL score of the associated MTAs, while the AEL of targets of the MTA with a negative AEL score is deducted from the AEL of the associated MTA. The AEL of the potential drivers whose aggregated score of the associations with the MTA are positive is added to the AEL score of the drug, while AEL of the potential drivers whose aggregated score of the associations with the MTA is negative are deducted from the AEL score of the MTA.

The final AEL score of the MTA is based on the number and evidence level of the associations between the MTA and all potential drivers and targets, and the AEL of the associated potential driver genes and druggable targets of the same patient. Consequently, AEL of the MTA is based on the aggregated score of multiple pieces of evidence that links the MTA to the whole molecular profile of the tumor, instead of assigning a rank to the MTA based on the highest available piece of evidence, which links the MTA to one driver.

### DDA based on the molecular profiles of patients treated in the SHIVA01 clinical trial

Version 1.64 of the system tested in this study connected 709 drivers and targets with 631 MTAs (registered and in clinical development). These drivers, targets, and MTAs were linked together with 12,620 connections (“if–then” rules), including 6089 clinical evidence and 6531 preclinical evidence-based connections deducted from 7306 publications.

In the randomized SHIVA01 trial, 11 MTAs were selected following a predefined treatment algorithm, molecular alterations–MTA pairs, based on clinically validated biomarker or supporting preclinical evidence. Of the 195 randomized patients, 170 were treated with MTAs based on SNVs in 50 genes and CNVs in 24 genes by NGS and expression level of three hormone receptors by immunohistochemistry (IHC) including patients after crossover from the chemotherapy treatment arm^15^. Both outcome data and complete molecular profiles were available for 113 patients^7^.

**Fig. 2:**
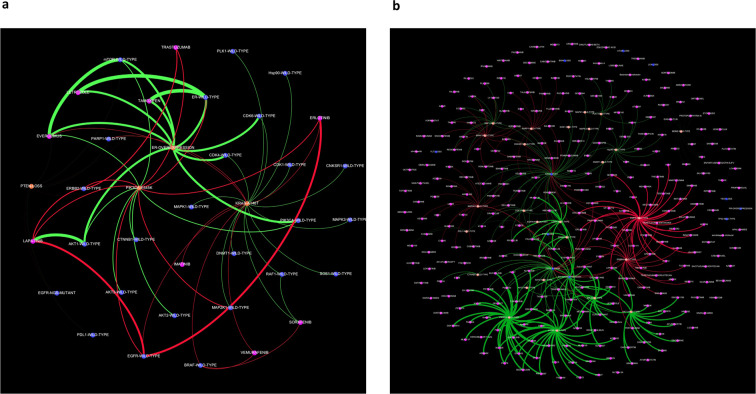
Network model of a real case from SHIVA01 analyzed by the digital drug-assignment (DDA) system. This drawing presents how drivers, targets, and MTAs are connected in the digital drug-assignment system in the case of patient “Example 5” (Tables 1 and 2 and Supplementary Table 2). Panel **a** depicts the connections of the 11 MTAs used in SHIVA01 to the drivers and targets in case of patient “Example 5”. Panel **b** depicts the connections of all MTAs in the database of the computational system to the drivers and targets in case of patient “Example 5”.

Molecular profiles of these 113 patients were uploaded into the DDA system. The system prioritized all driver alterations and identified associated targets and MTAs that are positive or negative connections with the molecular profiles of all patients. The system used multiple connections based on functional evidence (clinical, preclinical, and in silico) and frequency-based evidence for each patient. DDA identified, on average, 17 associated targets and 47 associated MTAs. DDA assigned an AEL score to all MTAs used to treat patients in the SHIVA01 trial (Supplementary Tables 1 and 2, Tables 1 and 2, and Fig. 2a, b).

**Table 1:** Digital drug assignment (DDA) of five examples cases of patients treated in the SHIVA01 trial.

Patient ID	Example 1	Example 2	Example 3	Example 4	Example 5
Tumor type	Colorectal cancer	Lung cancer	Colorectal cancer	Lung cancer	Breast cancer
Molecular profile	KRAS-G12V, TP53-A159V; PTEN loss	TP53-P278R, FLT3-M665T; INPP4B, STK11 loss; RICTOR amplification	APC-E1295del; PTEN, STK11, INPP4B loss	AR, ER expression; TP53-G266E; PTEN, STK11, INPP4B loss	ER, PR expression; PIK3CA-E545K, KRAS-A146T; PTEN loss
Molecular alteration-MTA pairing used in SHIVA01	PTEN loss-everolimus	FLT3 mutation-sorafenib	PTEN loss-everolimus	AR expression-abiraterone	PIK3CA mutation and PTEN loss-everolimus
AEL score of the MTA used	−1291.37	191.61	266.93	315.04	4165
Response to MTA	Progressive disease	Stable disease	Stable disease	Stable disease	Partial response
All targets (*n*)	25	16	9	22	25
Positive	20	15	6	19	20
Negative	5	1	3	3	6
All associated drugs (*n*)	271	226	200	269	287
Registered-positive	75	88	81	91	76
Registered-negative	33	10	17	17	31
Clinical development-positive	109	123	82	140	128
Clinical development-negative	54	5	20	21	65
All evidence used (*n*)	1894	1778	1138	1716	1165
Frequency-based	466	547	539	409	265
Function-based	871	689	491	769	765
Other	557	542	108	538	135
All connections (*n*)	2886	2498	1362	2450	1888
Clinical evidence-based	998	724	629	829	907
Preclinical evidence-based	1322	1142	178	1128	643
In silico evidence-based	25	27	3	21	11
Frequency-based	541	605	552	472	327

The tumor type, the molecular profile, the molecular alteration–molecularly targeted therapy (MTA) pairing used, the DDA AEL value of the MTA used, response to therapy in the SHIVA01, targets, MTAs associated with the molecular profile, and the number of evidence, and associations (“rules”) used for the analysis by the DDA system.

**Table 2:** Digital drug assignment (DDA) of a patient treated in the SHIVA01 trial.

Patient ID	Tumor type	Drivers (5)	AEL	Targets (25)	AEL	MTAs other compounds (287)	AEL
Example 5	Breast cancer	ER OVEREXPRESSION	731.820	PIK3CA WILD-TYPE	1251.37	PALBOCICLIB	4604.32
		PIK3CA-E545K	276.630	ER WILD-TYPE	1222.00	LETROZOLE	3898.00
		KRAS-A146T	150.910	mTOR WILD-TYPE	1102.61	ABEMACICLIB	3080.47
		PTEN LOSS	50.040	AKT1 WILD-TYPE	1013.79	RIBOCICLIB	3058.48
		PR OVEREXPRESSION	5.500	CDK4 WILD-TYPE	888.34	EVEROLIMUS	2886.78
				CDK6 WILD-TYPE	883.44	TAMOXIFEN	2773.34
				AKT2 WILD-TYPE	277.74	GSK2126458	2634.61
				CTNNB1 WILD-TYPE	277.07	DACTOLISIB	2632.83
				CTNNB1 WILD-TYPE	276.99	FULVESTRANT	2472.13
				RAF1 WILD-TYPE	154.78	VOXTALISIB	2354.17
				PLK1 WILD-TYPE	153.74	PWT33597	2353.98
				SOS1 WILD-TYPE	152.75	PI-103	2353.98
				HSP90 WILD-TYPE	152.62	VS-5584	2353.98
				MAPK1 WILD-TYPE	152.36	PKI179	2353.98
				MAPK3 WILD-TYPE	152.36	SF1126	2353.98
				CNKSR1 WILD-TYPE	151.76	GEDATOLISIB	2353.98
				DNMT1 WILD-TYPE	151.76	PF-04691502	2353.98
				CDK1 WILD-TYPE	151.21	P7170	2353.98
				PDL-1 WILD-TYPE	50.52	APITOLISIB	2353.98
				EGFR MUTANT	−50.73	DS-7423	2353.98
				PARP1 WILD-TYPE	−101.56	BGT226	2353.98
				MAP2K1 WILD-TYPE	−115.93	TASELISIB	2287.02
				BRAF WILD-TYPE	−158.39	ALPELISIB	2268.62
				ERBB2 WILD-TYPE	−334.38	AZD9496	2040.32
				EGFR WILD-TYPE	−675.79	GDC-0810	2008.22
						ELACESTRANT	1997.07
						EXEMESTANE	1984.30
						RONICICLIB	1922.99
						IPATASERTIB	1569.74
						GDC-0077	1538.00
						PAXALISIB	1531.18
						PICTILISIB	1529.93
						BUPARLISIB	1529.02
						XL147	1528.95
						COPANLISIB	1528.55
						TEMSIROLIMUS	1447.09
						SIROLIMUS	1388.14
						and additional 236 compounds between 1389 and -739 AELs	
						TRASTUZUMAB	−738.57
						GEFITINIB	−841.29
						NERATINIB	−1010.01
						EPERTINIB	−1010.17
						TAK-285	−1010.17
						PELITINIB	−1010.17
						CUDC-101	−1010.17
						AV-412	−1010.17
						ALLITINIB	−1010.17
						AFATINIB	−1060.72
						CETUXIMAB	−1110.83
						ERLOTINIB	−1130.73
						DACOMITINIB	−1215.03
						PANITUMUMAB	−1487.93

The DDA scores of drivers, targets, and associated MTAs of a case “Patient ID example 5”.

### Association between the DDA AEL scores and the disease control rates of MTAs used in the SHIVA01 trial

**Fig. 3:**
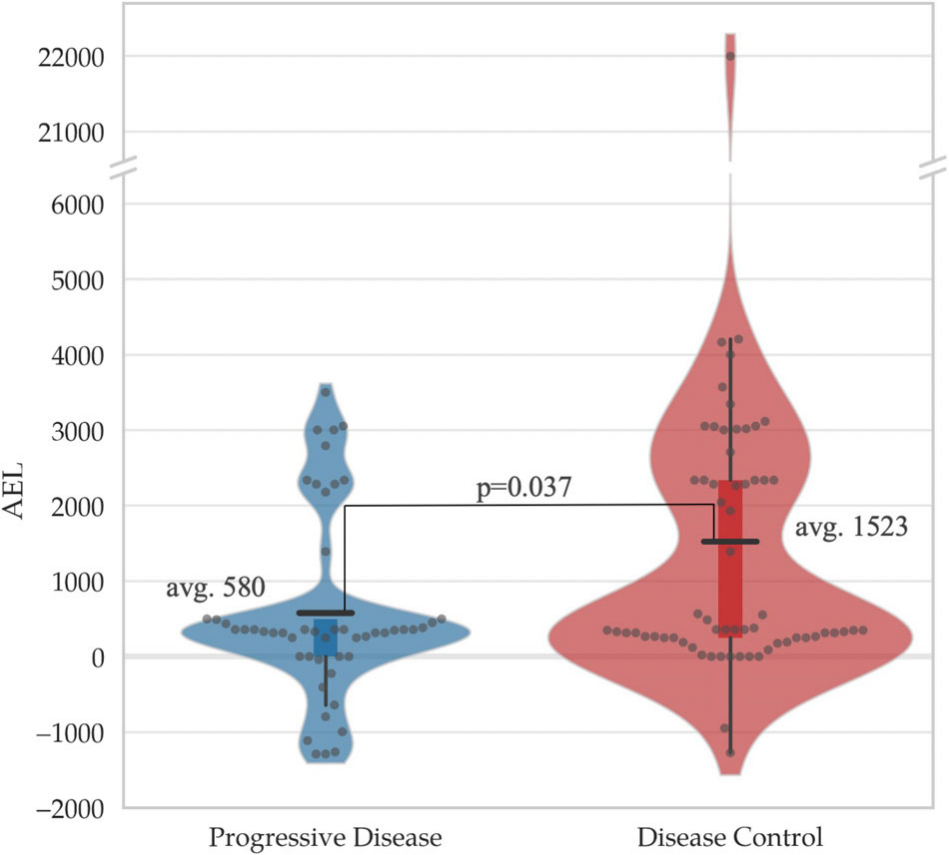
Association between digital drug assignment (DDA) and disease control of molecularly targeted agents (MTAs) in the SHIVA01 trial. Average aggregated evidence level (AEL) score of MTAs in patients with progressive disease and patients achieving disease control (DCR) (stable diseased and partial response).

The calculated average AEL scores of the employed MTAs were around threefold higher in case of patients achieving disease control (partial response (PR) and stable disease (SD)) than in non-responders in the SHIVA01 trial^7,15–17^, and the difference was significant (1523 versus 580, *P* = 0.037) (Fig. 3).

**Fig. 4:**
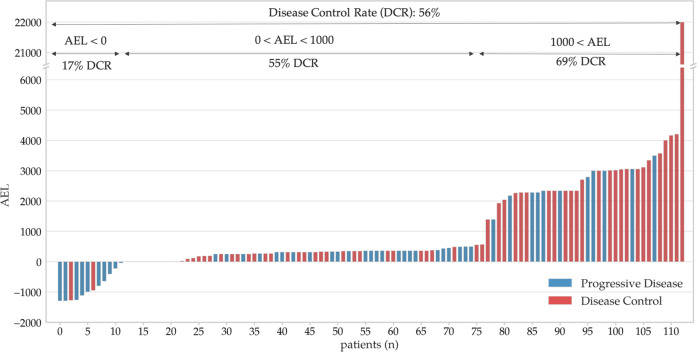
Distribution of AEL values of molecularly targeted agents (MTAs) assigned by the digital drug assignment (DDA) used in the SHIVA01 trial. DDA AEL scores of MTAs used in SHIVA01 patients with progressive disease (PD) (blue bars) and patients achieving DCR (red bars).

Molecularly targeted drugs used in the SHIVA01 were arbitrarily classified into three groups according to their DDA AEL scores into low (AEL < 0), intermediate (0 < AEL < 1000), and high (1000 < AEL) DDA tiers (Fig. 4). The cut-off for the high DDA tier was chosen based on the distribution histogram of AEL values blind to the outcome data (Supplementary Fig. 1). In the low tier (*n* = 12, 11%), the calculated AEL values were negative, indicating that the calculated evidence level of associations predicting resistance (negative drug relations) was higher than the AEL of positive associations. In the intermediate (*n* = 65, 57%) and high tiers (*n* = 36, 32%), the AEL of positive associations was higher than the AEL of negative associations. The disease control rate (DCR) (PR and SD) was 56% in the total patient population, 17% in the low, 55% in the intermediate, and 69% in the high DDA tiers, respectively.

### Association between the DDA AEL scores, progression-free survival (PFS), and overall survival (OS) of MTAs used in the SHIVA01 trial

**Fig. 5:**
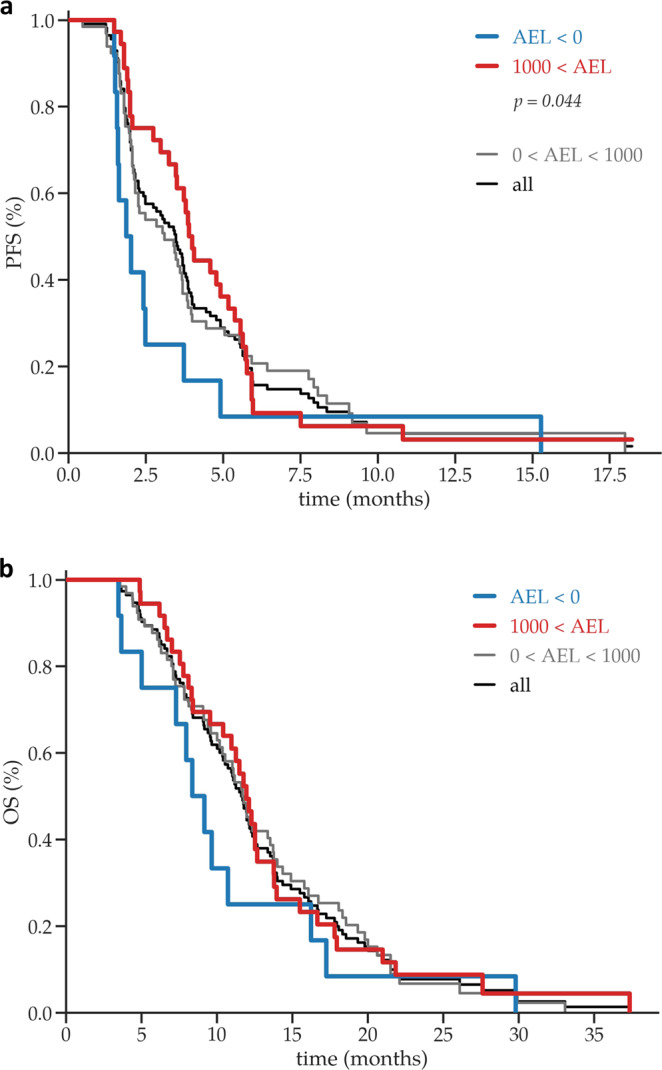
Progression-free survival (PFS) and overall survival (OS) of patients treated with molecularly targeted agents (MTAs) in different digital drug-assignment (DDA) AEL score tiers. Kaplan–Meier estimates of **a** progression-free survival (PFS) and **b** overall survival (OS) of patients treated with matched molecularly targeted therapy in SHIVA01 according to DDA AEL tiers.

The PFS and OS of the DDA tiers were compared using log-rank tests (Fig. 5). The median PFS of the whole population was 3.48 months (*n* = 113). The median PFS was significantly longer in patients treated with drugs of high AEL (1000 < AEL) than in the low AEL (AEL < 0) DDA tier (3.95 versus 1.95 months, *P* = 0.044); hazard ratio: (p(AEL < 0)/p(AEL > 1000)): 1.91 (95% CI 0.86–4.23) and (p(AEL > 1000)/p(AEL < 0)): 0.52 (95% CI 0.24–1.16). The median PFS of the intermediate tier (0 < AEL < 1000) was 3.11 months (Fig. 5a). To further evaluate the relationship between the PFS and the AEL scores of MTAs patients were grouped according to PFS by above and below the median PFS of 3.48 months, there is a statistically significant difference in the average AEL values of the two groups: 1625.9 (above median PFS) versus 611.9 (below median PFS) (*P* = 0.02336).

The median OS of the whole population was 11.15 months and the median OS of the intermediate tier (0 < AEL < 1000) was 11.63 months (Fig. 5b). Patients in the SHIVA01 trial were allowed to crossover between the MTA and chemotherapy arms, limiting the evaluation of the OS results. However, there was a trend of patients having worse OS in the low DDA tier; the difference did not reach statistical significance (8.78 months versus 12.09 months).

### Concordance between the DDA-based therapy recommendations and MTAs chosen in the SHIVA01 trial

**Fig. 6:**
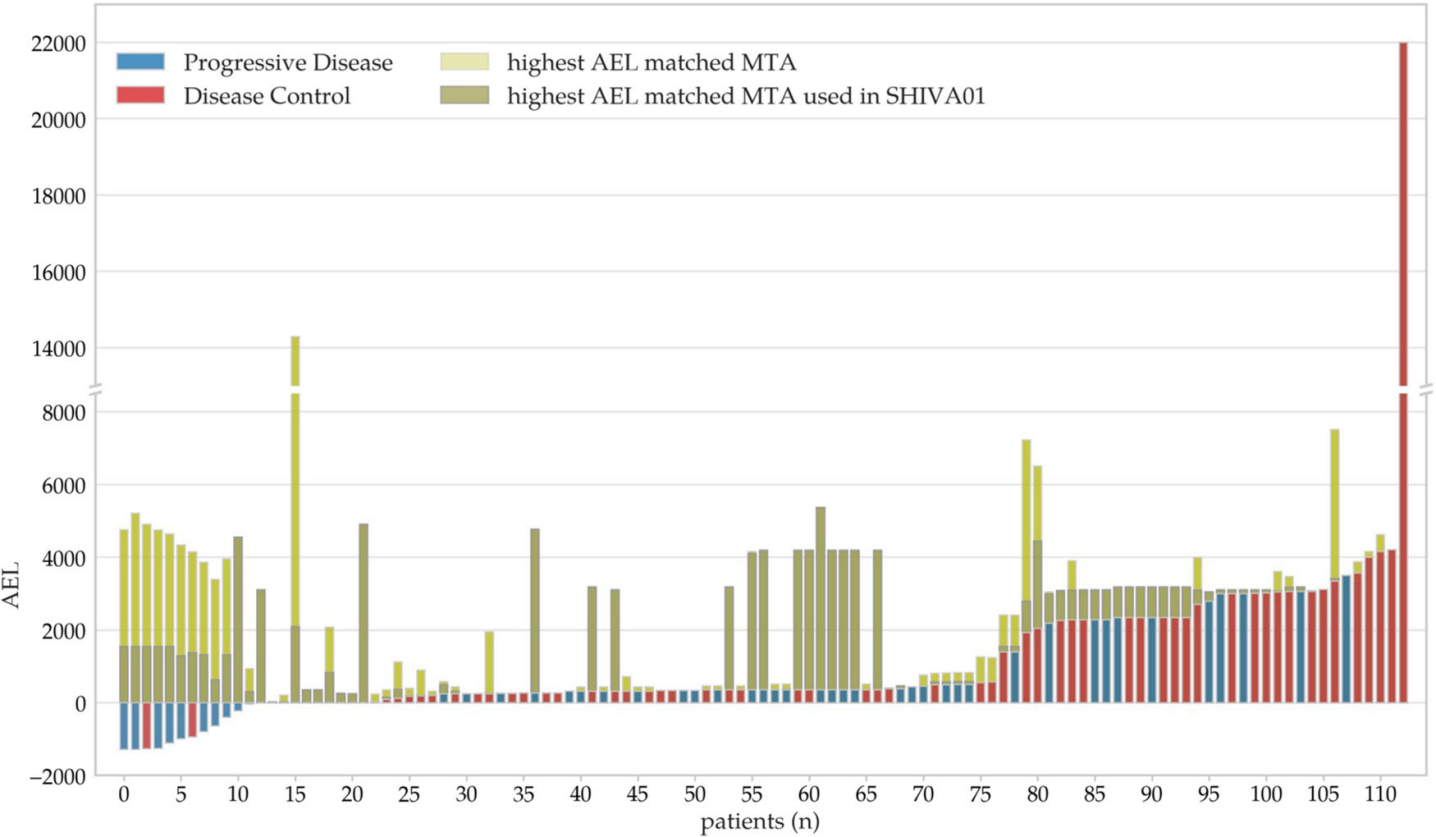
Distribution of MTAs with the highest AEL values assigned by the digital drug-assignment (DDA) for patients in the SHIVA01 trial. In addition to DDA AEL scores of MTAs used in SHIVA01 patients with progressive disease (PD) (blue bars) and patients achieving DCR (red bars). The DDA AEL levels of MTAs with the highest AEL values assigned by the DDA out of the 11 MTAs used in the SHIVA01 trial (dark green bars) and out of all 631 MTAs in the current database of the DDA system (light green bars).

DDA would assign the highest AEL score to the same MTA used in SHIVA01 in 60 patients (53%) and assign a different MTA in 53 patients (47%) out of the 11 drugs used in the SHIVA01 trial. If the trial was today, DDA would still assign the highest AEL score to the same MTA in 28 patients (25%) and would assign a different MTA for 85 patients (75%) out of the 631 MTAs (registered and under development) available today in the system’s current database. For simplicity, letrozole and tamoxifen were grouped for this analysis (Fig. 6). DDA was more likely to assign the highest AEL to the same drug for patients in the higher DDA tiers (DDA Tier I: 0/12 (0%), DDA Tier II: 13/65 (20%), DDA Tier III: 15/36 (42%)) from the currently available 631 MTAs. In cases where patients responded to the MTA, the AEL score of the highest ranking MTA by the DDA out of the MTAs available today is the same or very similar to the AEL score of the MTA used in the SHIVA01.

## Discussion

Here we report a computational method, DDA, to prioritize MTAs for precision oncology. DDA is an “open box,” explainable AI system. The rationale and mechanism of action are described here in detail, and the concrete algorithm and the whole evidence database used for each case can be retrieved and reviewed for human supervision for quality assurance purposes. The system is a rule-based expert system that contains a large number of if–then relations and algorithms. This type of device is in the scope of the Artificial Intelligence/Machine Learning (AI/ML)-Based Software as a Medical Device (SaMD) Action Plan of the FDA^14^.

We used the molecular information and outcome data of patients treated with MTAs in the SHIVA01 trial for the clinical performance analysis of the DDA-based software system, the Realtime Oncology Treatment Calculator^TM^. It is important to note that the data of SHIVA01 were not used to train or optimize the AI system. This is important because this excludes the possibility of “overfitting,” the common issue of AI systems. The patients’ molecular profiles were uploaded into the software system, and the AEL scores MTAs associated with the profiles were calculated blindly to the outcome data prospectively on the retrospective data. Next, the clinical benefit of MTAs with different AEL scores was analyzed in different patient groups according to the treatment outcomes. There was a statistical difference between the AEL scores of MTAs according to the disease control and below or above median PFS. In addition, the average PFS was significantly longer in patients treated with drugs of high AEL (1000 < AEL) than in the low AEL (AEL < 0) DDA tiers. The thresholds for these tiers were arbitrary, selected based on the distribution of AELs (Supplementary Fig. 1). For example, setting the threshold at 500 increases the DCR to 71% in the high tier. Similarly, the Kaplan–Meier estimation is also slightly more significant when the high AEL is defined as >500 (*P* = 0.042) than with >1000 group (*P* = 0.044). There was a case with BRAFV600E mutation with an outlying high AEL value. If we omitted this case from the analysis, the difference between the AEL values of the DCR and PD group would have been even more significant (*P* = 0.015 versus *P* = 0.037).

There were several advantages of using the data from the SHIVA01 for the clinical performance analysis of the DDA system. In this clinical trial, the same standard molecular diagnostic tests were performed for all patients, and the outcome was assessed by the same methodology. The patients represented a relevant distribution of different types of advanced solid cancers. SHIVA01 was a prospective trial. Therefore, there was no bias toward reporting only the outstanding responders like in case studies.

The limitations of this study are that patients included in the SHIVA01 were heavily pretreated, which limited the assess of the full potential of MTAs. The focused 50 genes NGS panel used in the trial is still widely used in molecular diagnostics today. This panel was designed to detect the most frequent driver alterations (“hot spots”) in epithelial cancers primarily included in this trial. Therefore, it was sufficient to identify the expected 3–4 driver alterations per tumor. However, the possibility of choosing from more MTAs than the 11 MTAs used in SHIVA01, based on comprehensive molecular profiling (300–600 gene panels, WES or WGS) covering all potential driver alterations, would more likely increase than decrease the clinical performance of DDA.

Using combinations of two or three therapies is also a logical solution to fight the complexity of cancer. The I-PREDICT trial and real-world data show that combination therapies covering more than half of the drivers overall achieve superior results than monotherapies covering less than half of drivers^12,18^. However, combination therapies also carry the disadvantage of higher risk of toxicity and further increase of the targeted therapies’ financial burden.

In SHIVA01, MTAs were mainly used in monotherapies targeting predefined molecular alterations. The average number of driver alterations in the three DDA tiers was very similar (3.92 in the low, 3.26 in the intermediate, and 3.73 in the high DDA tier). Thus, DDA AEL scores of MTAs in monotherapy correlated with better clinical outcomes independently from the number of concurrent driver alterations in this study.

These results indicate that the DDA system can identify MTAs that are more likely to be effective despite unmatched driver alterations. This outcome can be achieved due to the network analysis system design of DDA. DDA identifies the MTA which inhibits the driver alteration(s) or indirect druggable molecular target(s), which have the highest number and level of positive associations—and least negative associations—with the highest number of all driver alterations of the same tumor (Figs. 1 and 2a, b).

The potential clinical utility of the current version of DDA based on the data presented here confines to the support of relative prioritization between MTAs in the absence of any randomized clinical trial evidence that would indicate the superiority of one MTA over the other. In SHIVA01, all MTAs were used in the presence of a predefined target chosen by the investigators’ molecular tumor board. MTAs associated with registered companion diagnostics (Level1/Tier I evidence by ESCAT) were excluded. The efficacy of MTAs used in the study was not different according to the lower ESCAT tiers^9^. Consequently, data presented in this study imply that choosing an MTA based on the higher DDA score will more likely lead to the same or better clinical benefit rather than worse in comparison to randomly choosing another MTA with a DDA lower score at the same evidence level.

In clinical practice, the DDA score can be useful if multiple MTAs are available at the same evidence levels. MTAs at the same evidence level should be registered in the same indication at the same treatment line, associated with the same companion diagnostic (ESCAT Tier I) molecular genetic alterations, or associated with multiple companion diagnostic alterations present in the same tumor or not linked to any companion diagnostic tests. DDA can also be useful when there are no registered treatment options available, but multiple MTAs in clinical use or clinical trials match molecular genetic alterations present in the patient’s tumor at the same evidence level. The ultimate goal of DDA is to help the work of molecular tumor boards planning of the personalized treatment strategy of all treatment lines for each patient. We provide three clinical examples for the clinical utility in the supplementary information (Supplementary Note 1).

It is important to note that the current DDA version is not intended to replace any on-label therapies with an MTA not registered in the same indication or at a higher clinical evidence level, based on the DDA score alone. We will need further retrospective and prospective clinical trials to evaluate the absolute clinical benefit of MTAs according to their DDA score, and we will need randomized trials to introduce new treatment options based on DDA in the registered treatment protocols. MTAs and corresponding DDAs can be co-developed and co-registered in the future. Based on data presented here, DDA can be potentially superior as a companion diagnostic method over most single biomarkers identifying patients who most benefit from an MTA, leading to the acceleration, reduced cost, and risk of drug development.

The DDA system can be used by physicians, pathologists, molecular biologists, or other professionals with relevant biomedical backgrounds who are specifically trained for the system’s utility and limitations. DDA is designed to be open for easy human supervision. The system generates the lists of published evidence used for the DDA score calculations and generates text descriptions of the information available on the molecular alterations and associated MTAs. DDA is linked to an online case management system designed for dynamic decision support for precision oncology. DDA can be updated as new molecular diagnostic test results are available for the patient. The system combines all available results from different laboratories and types of tests: NGS, fluorescent in situ hybridization (FISH), IHC (for example, ER, PDL-1), TMB, and MSI to generate a prioritized list of MTAs associated with all these results combined.

An essential feature of DDA that it is based on a continuously expanded extensive database of evidence-based associations between driver genes, targets, and MTAs. Therefore, DDA mirrors the improvement of our understanding of cancer biology and the availability of novel molecularly targeted treatments over time. As a result, the AEL scores of the same MTAs and drug assignments for the same patient can change over time. The other important feature of the system is that since the targets of MTAs are known by definition, experience (preclinical and clinical) with MTAs in the presence of different driver mutations increases our understanding of the functional relevance of specific mutations and teaches about the associations between drivers and druggable targets. This further teaches the network, which can better and better predict the outcome of any future MTA without previous experience based on the functional significance of its targets.

Since DDA uses standardized evidence databases and algorithms to aggregate evidence-based associations, the predictive performance of the computational system and the AEL score can be continuously tested on test databases and on real-world experience to ensure that the performance is reproducible, and the new version of the system is at least as good or better in predicting response to MTAs. Regarding this point, we have reproduced the analysis presented in this report with a newer 1.67 version of the Realtime Oncology Treatment Calculator^TM^ and found the same or slightly better results (data not shown).

The use of AI in precision medicine, especially in precision oncology, is highly anticipated^19^. The FDA has initiated public discussion on this subject and created an action plan on how to regulate AI-based software as medical devices (SaMD)^14^. The definition of AI used by the FDA is any “intelligent” software. AI tools transform information by algorithms to improve diagnostic or treatment decisions. Algorithms in AI-assisted treatment assignment algorithms can be developed manually as in the current version of the DDA or using ML on training datasets. A National Cancer Policy Forum has already reviewed this emerging field: Improving Cancer Diagnosis and Care: Clinical Application of Computational Methods in Precision Oncology^20^.

It is important to note that most software solutions currently used to interpret NGS results are reference tools. These systems are designed to store and retrieve information from structured databases and guidelines to automatize interpretation for electronic reporting. The expected performance of these reference tools is to help users to reach the same conclusion they would reach anyway, but faster and more conveniently. These systems are based on large databases of single gene/biomarker—MTA parings prioritized based on the evidence level of the evidence selected by a team of experts who perform the literature search and curation^21^. Some of these systems use AI for text recognition (natural language processing, NLP) to automatize the medical literature search before human curation, but this does not mean that they also use AI-based drug-assignment algorithms^22^.

There are ongoing efforts to develop more complex decision support systems. The “simplified interventional mapping system” (SIMS) developed by Worldwide Innovative Networking (WIN) consortium offers a systematic approach for the prioritization of druggable targets (“interventional points”) using a scoring system, which reflects the type and extent of changes in genes connected to a group of targets^23^. The approach was tested in the WINTHER trial^24^. Results indicated that besides the “matching score” of the DNA alterations, the ranking of targets using the WINTHER algorithm calculating the extent of mRNA expression in the tumor compared to the corresponding normal tissue also correlated with the clinical benefit of MTAs^24^. The WINTHER trial results also support the prioritization of targets using mathematical algorithms based on the complex biology of cancer as predictive diagnostic tools instead of using single biomarkers.

PreciGENE^TM^ (CureMatch Inc., San Diego, CA, USA) primarily focusing on the identification of effective combination therapies is an important example of rule-based AI-assisted computational tools like DDA with the Realtime Oncology Treatment Calculator^TM^^25^.

We expect that more and more solutions similar to these systems will emerge in the next few years. We also expect that large randomized clinical trials will compare different AI-based treatment assignment algorithms against another^26,27^. The goal will be to find the right predictive AI algorithm for every MTA in each indication. AI systems will never “make decisions.” The predictive scores generated by these systems will eventually help us make better treatment decisions in the next, digital age of precision oncology.

## Methods

### Data collection and analysis

The software of the DDA system used in this study was the Realtime Oncology Treatment Calculator^TM^ version 1.64. PFS and OS data were represented by Kaplan–Meier estimation and the survival end points were compared using log-rank tests. Hazard ratio was computed by log-rank method. *T*-test was used to determine the significance of difference between the means of groups. Statistical analysis was performed using the NumPy^28^, SciPy^29^, and lifelines^30^ modules of Python 3.7.

### Human research

The study was conducted in accordance with the Declaration of Helsinki, and the protocol was approved by the Ethics Committee of the National Institute of Pharmacy and Nutrition (approval ID: OGYEI/50268/2017). The SHIVA01 clinical trial was approved by the Ile-de-France ethics committee and informed consent was obtained from all human participants. The trial was carried out in accordance with the Declaration of Helsinki, the Good Clinical Practice guidelines of the International Conference on Harmonization, and relevant French and European laws and directives.

### Ethical compliance

During the preparation, submission, conduct, and analysis of this study, we complied with all relevant ethical regulations.

### Reporting summary

Further information on research design is available in the Nature Research Reporting Summary linked to this article.

## Supplementary information

Supplementary Information

Supplementary Data 1

Supplementary Data 3

Supplementary Data 4

Supplementary Data 5

Supplementary Data 6

Supplementary Data 7

Supplementary Data 2

Supplementary Data 8

Supplementary Data 9

Supplementary Data 10

Reporting Summary

## Data Availability

The data generated and analyzed during this study are described in the following data record: 10.6084/m9.figshare.14414612 (ref. ^31^). The data are available within the paper and/or uploaded in reusable format to the figshare repository, and are openly available at 10.6084/m9.figshare.14331323 (ref. ^32^).

## Code availability

RealTime Oncology Treatment Calculator™ code is proprietary to Oncompass Medicine Inc. and it is available for collaborative purposes upon request.

## References

[1] Peták I, Schwab R, Orfi L, Kopper L, Kéri G (2010). Integrating molecular diagnostics into anticancer drug discovery. Nat. Rev. Drug Discov..

[2] Le Tourneau C, Borcoman E, Kamal M (2019). Molecular profiling in precision medicine oncology. Nat. Med..

[3] Flaherty, K. T. et al. NCI-MATCH Team. Molecular landscape and actionable alterations in a genomically guided cancer clinical trial: National Cancer Institute Molecular Analysis for Therapy Choice (NCI-MATCH). *J. Clin. Oncol.* **38**, 3883–3894 (2020). 10.1200/JCO.19.03010 PMC7676882 33048619

[4] Mangat, P. K. et al. Rationale and design of the Targeted Agent and Profiling Utilization Registry (TAPUR) Study. *JCO Precis. Oncol*. **2018**10.1200/PO.18.00122 (2018). 10.1200/PO.18.00122 PMC6312096 30603737

[5] Tsimberidou AM (2019). Long-term overall survival and prognostic score predicting survival: the IMPACT study in precision medicine. J. Hematol. Oncol..

[6] Massard C (2017). High-throughput genomics and clinical outcome in hard-to-treat advanced cancers: results of the MOSCATO 01 trial. Cancer Discov..

[7] Le Tourneau C (2015). Molecularly targeted therapy based on tumour molecular profiling versus conventional therapy for advanced cancer (SHIVA): a multicentre, open-label, proof-of-concept, randomised, controlled phase 2 trial. Lancet Oncol..

[8] Mateo J (2018). A framework to rank genomic alterations as targets for cancer precision medicine: the ESMO Scale for Clinical Actionability of molecular Targets (ESCAT). Ann. Oncol..

[9] Moreira A (2019). Efficacy of molecularly targeted agents given in the randomised trial SHIVA01 according to the ESMO Scale for Clinical Actionability of molecular Targets. Eur. J. Cancer.

[10] Tate JG (2019). COSMIC: the catalogue of somatic mutations in cancer. Nucleic Acids Res..

[11] ICGC/TCGA Pan-Cancer Analysis of Whole Genomes Consortium. (2020). Pan-cancer analysis of whole genomes. Nature.

[12] Sicklick JK (2019). Molecular profiling of cancer patients enables personalized combination therapy: the I-PREDICT study. Nat. Med..

[13] McCarthy D (2007). Computers getting the drift. Philos. Trans. A Math. Phys. Eng. Sci..

[14] U.S. Food and Drug Administration. *Artificial Intelligence/Machine Learning (AI/ML) Software as a Medical Device Action Plan* (U.S. Department of Health and Human Services, 2021).

[15] Belin L (2017). Randomized phase II trial comparing molecularly targeted therapy based on tumor molecular profiling versus conventional therapy in patients with refractory cancer: cross-over analysis from the SHIVA trial. Ann. Oncol..

[16] Kamal M (2018). Revisited analysis of a SHIVA01 trial cohort using functional mutational analyses successfully predicted treatment outcome. Mol. Oncol..

[17] Servant N (2014). Bioinformatics for precision medicine in oncology: principles and application to the SHIVA clinical trial. Front. Genet..

[18] Kato S (2020). Real-world data from a molecular tumor board demonstrates improved outcomes with a precision N-of-One strategy. Nat. Commun..

[19] Panagiotou OA (2020). Clinical application of computational methods in precision oncology: a review. JAMA Oncol..

[20] National Academies of Sciences, Engineering, and Medicine; Health and Medicine Division; Board on Health Care Services; National Cancer Policy Forum. Improving cancer diagnosis and care: clinical application of computational methods in precision oncology, *Proc. Workshop* (eds. Nass, S. J. et al.) (National Academies Press, 2019). 31386317

[21] Chakravarty, D. et al. *OncoKB: A Precision Oncology Knowledge Base*. *JCO Precis Oncol*. **2017**10.1200/PO.17.00011 (2017). 10.1200/PO.17.00011 PMC5586540 28890946

[22] Itahashi K (2018). Evaluating clinical genome sequence analysis by Watson for genomics. Front. Med. (Lausanne).

[23] Lazar V (2015). A simplified interventional mapping system (SIMS) for the selection of combinations of targeted treatments in non-small cell lung cancer. Oncotarget.

[24] Rodon J (2019). Genomic and transcriptomic profiling expands precision cancer medicine: the WINTHER trial. Nat. Med..

[25] Boichard A, Richard SB, Kurzrock R (2020). The crossroads of precision medicine and therapeutic decision-making: use of an analytical computational platform to predict response to cancer treatments. Cancers (Basel).

[26] Mittra A, Moscow JA (2019). Future approaches to precision oncology-based clinical trials. Cancer J..

[27] Topol EJ (2019). A decade of digital medicine innovation. Sci. Transl. Med..

[28] Harris CR (2020). Array programming with NumPy. Nature.

[29] Virtanen, P. et al. and SciPy 1.0 Contributors. SciPy 1.0: Fundamental algorithms for scientific computing in Python. *Nat. Methods* **17**, 261–272 (2020). 10.1038/s41592-019-0686-2 PMC7056644 32015543

[30] Davidson-Pilon, C. et al. CamDavidsonPilon/lifelines: v0.24.8 (Version v0.24.8). Zenodo. 10.5281/zenodo.3833188 (2020).

[31] Petak, I. et al. Metadata record for the manuscript: a computational method for prioritizing targeted therapies in precision oncology: performance analysis in the SHIVA01 trial. figshare 10.6084/m9.figshare.14414612 (2021). 10.1038/s41698-021-00191-2 PMC8222375 34162980

[32] Petak, I. et al. Datasets for the article: A computational method for prioritizing targeted therapies in precision oncology: performance analysis in the SHIVA01 trial. figshare 10.6084/m9.figshare.14331323 (2021). 10.1038/s41698-021-00191-2 PMC8222375 34162980

