# Supplementary material for: A computational method for prioritizing targeted therapies in precision oncology: performance analysis in the SHIVA01 trial
Source: NPJ Precis Oncol. 2021 Jun 23;5:59. doi: 10.1038/s41698-021-00191-2 (PMC8222375; doi:10.1038/s41698-021-00191-2)
Supplement: Supplementary file 1 — Supplementary Information [file 41698_2021_191_MOESM1_ESM.pdf]

SUPPLEMENTAL INFORMATION

Supplementary Table 1. Tumor sites, histology, molecular profile, molecularly targeted therapy used in the SHIVA01 trial, AEL score assigned by the Digital Drug-Assigment system (DDA), best response, PFS, OS

Supplementary Table 1. Detailed clinical and molecular information of patients treated with MTAs in SHIVA01 trial

SHIVA ID	Primary tumor	Histology type	Molecular profile	Therapy in trial	AEL of drug applied in trial	Best response	PFS	OS
01-0001	head-neck	squamous cell carcinoma	STK11 loss; AKT1, AKT2, PIK3CB, RICTOR ampl	everolimus	22.13	SD	3.44	6.31
01-0004	ovary	adenocarcinoma	JAK3-V722I, ATM-K1698T; AR, ESR1, PGR exp	tamoxifen	2336.93	SD	5.44	10.84
01-0010	cervix	adenocarcinoma	ESR1 exp	letrozole	3116.53	SD	3.9	17.81
01-0017	breast	UNKNOWN	AR, ESR1, PGR exp	letrozole	4205.31	SD	5.93	37.36
01-0018	breast	UNKNOWN	PIK3CA-H1047R	everolimus	1391.17	PD	1.48	12.29
01-0025	breast	UNKNOWN	PTPN11-E69K, TP53-E286K; AR exp	abiraterone	357.46	PD	1.7	6.18
01-0039	breast	UNKNOWN	AR, ESR1, PGR exp	abiraterone	357.46	SD	1.64	13.73
01-0041	breast	UNKNOWN	AR, ESR1, PGR exp; PTEN loss	abiraterone	357.46	PD	2	11.7
01-0044	UNKNOWN	sarcoma	PGR exp	tamoxifen	0.23	PD	2.07	29.9
01-0057	lung	UNKNOWN	TP53-R249G, EGFR-746_751del; STK11 loss; HER2 ampl	lapatinib + trastuzumab	2042.81	SD	2	4.9
01-0062	anus	squamous cell carcinoma	PIK3CA-E545K	everolimus	489.67	PD	3.7	13.54
01-0064	lung	UNKNOWN	STK11-D194Y, TP53-V173L; STK11 loss	everolimus	88.3	SD	1.25	11.99
01-0070	ovary	adenocarcinoma	TP53-C242Y; ESR1, PGR exp	tamoxifen	2336.93	SD	10.82	11.76
01-0073	ovary	adenocarcinoma	TP53-Y234C, KDR-G1308R; AR, ESR1 exp	abiraterone	348.14	SD	3.87	15.8
01-0078	lung	UNKNOWN	TP53-R249M, APC-Q1447fs; PTEN loss	everolimus	249.45	PD	5.05	11.79
01-0081	endometrium	adenocarcinoma	TP53-F270C, TP53-Y126_S127delins*; AR, PGR exp	abiraterone	348.14	PD	1.67	20.63
01-0091	lung	UNKNOWN	KRAS-G13C; ESR1 exp	tamoxifen	2284.13	SD	3.48	7.03
01-0099	breast	UNKNOWN	ESR1, AR, PGR exp	abiraterone	357.46	SD	7.77	33.08
01-0114	UNKNOWN	urothelial carcinoma	PIK3CA-T1025A, TP53-R175H; ESR1 exp; PTEN loss; RICTOR ampl	everolimus	3047.74	SD	4	7.56
01-0115	breast	UNKNOWN	AR, ESR1, PGR exp; STK11, INPP4B loss	abiraterone	357.46	PD	1.8	3.58
01-0119	UNKNOWN	adenocarcinoma	KRAS-Q61H; SKT11, PTEN loss	everolimus	-39.98	PD	2.49	17.25
01-0122	skin	malignant melanoma	BRAF-N581T; INPP4B, PTEN loss	everolimus	249.45	PD	1.8	7
01-0124	UNKNOWN	adenoid cystic carcinoma	PDGFRA, KIT ampl	sorafenib	0.49	SD	5.64	16.07
01-0126	head-neck	squamous cell carcinoma	ESR1 exp; YES1 ampl	tamoxifen	2284.13	PD	1.9	10.41
01-0139	lung	UNKNOWN	STK11, PTEN loss	everolimus	266.8	SD	2.03	13.77
01-0148	colon	adenocarcinoma	KRAS-G12V, PIK3CA-E542K	everolimus	-1113.66	PD	3.74	10.74
01-0158	UNKNOWN	adenoid cystic carcinoma	PIK3CA-E545K, TP53-V172F	everolimus	486.02	SD	5.7	19.35
01-0160	head-neck	squamous cell carcinoma	ESR1 exp; SRC ampl; PTEN loss	everolimus	3002.26	SD	5.38	10.97
01-0162	ovary	adenocarcinoma	TP53-V157G; ESR1, PGR exp; STK11, PTEN loss	everolimus	3054.15	PD	2.98	13.77
01-0166	gastroesophageal junction	adenocarcinoma	TP53-G266E; PTEN, STK11, INPP4B loss, HER2, SRC ampl	everolimus	266.54	PD	1.25	3.98
01-0172	head-neck	squamous cell carcinoma	TP53-W91; STK11 loss; EGFR ampl	erlotinib	552.65	SD	3.67	9.59
01-0181	cervix	squamous cell carcinoma	PIK3CA-E545K	everolimus	498.45	PD	1.97	10.02
01-0182	colon	adenocarcinoma	KRAS-G12V, TP53-G266E; PTEN, INPP4B, STK11 loss	everolimus	-1274.84	SD	4.92	16.23
01-0183	gastroesophageal junction	adenocarcinoma	TP53-R282W; HER2 ampl, FLT3 ampl	imatinib	1.5	PD	1.38	7.1
01-0191	colon	adenocarcinoma	KRAS-G12A, PIK3CA-E545K	everolimus	119.35	SD	3.97	7.85
01-0197	head-neck	squamous cell carcinoma	TP53-R248Q; PTEN loss	everolimus	249.74	PR	9.08	14.92
01-0206	colon	adenocarcinoma	KRAS-G12V, PTEN-L23*, TP53-E180K; PTEN loss	everolimus	-1263.78	PD	1.61	7.98
01-0222	ovary	adenocarcinoma	ESR1 exp; STK11 loss	tamoxifen	2336.93	PD	1.7	6.54
02-0002	endometrium	sarcoma	PTEN loss, PDGFRA ampl	sorafenib	0.64	SD	1.21	4.44
02-0017	cervix	squamous cell carcinoma	FGFR1-K139E; ESR1, PGR exp; STK11 loss, EGFR ampl	erlotinib	566.58	PR	9.64	18.07
02-0035	liver	hepatocellular carcinoma	AR exp	abiraterone	315.04	SD	7.93	18.56
02-0046	UNKNOWN	neuroendocrine tumor (NET)	ESR1 exp; PTEN, STK11 loss	dasatinib	0	SD	8.92	20.3
02-0057	colon	adenocarcinoma	KRAS-G12D, TP53-R156G, ERBB2-S792F; FLT3 ampl	lapatinib + trastuzumab	-407.56	PD	1.87	8.38
02-0071	lung	UNKNOWN	KRAS-G12A; ESR1 exp; STK11, PTEN loss; PIK3CB ampl	everolimus	2708.03	SD	3.25	7.79
02-0072	salivary gland	adenocarcinoma	PIK3CB ampl; PTEN loss	everolimus	250.01	SD	5.54	21.52
02-0078	head-neck	squamous cell carcinoma	ESR1 exp; PTEN, INPP4B loss	everolimus	3002.26	PD	2	8.11
02-0083	gastroesophageal junction	adenocarcinoma	PIK3CA-H1047Y, TP53-R248W, RET-E621K; INPP4B loss	everolimus	188.87	SD	3.11	9.4
02-0086	liver	hepatocellular carcinoma	ESR1 exp; PTEN, STK11 loss	everolimus	3018.5	SD	5.97	13.96
03-0002	lung	UNKNOWN	TP53-G266E; ESR1, AR exp; PTEN, STK11, INPP4B loss	abiraterone	315.04	SD	3.61	11.04
03-0015	UNKNOWN	sarcoma	SMO-S205fs, TP53, PTEN loss	everolimus	249.45	SD	2.1	12.35
03-0023	ovary	granulosa cell tumor, mal.	ATM-V410A; ESR1, PGR, AR exp	abiraterone	315.04	SD	7.54	13.73
04-0005	breast	UNKNOWN	KRAS-G12V, PIK3CA-H1047R; ESR1, PGR loss	everolimus	3344.74	SD	4.92	6.21
04-0007	breast	UNKNOWN	AR, ESR1, PGR exp	abiraterone	357.46	SD	3.05	4.73
04-0008	UNKNOWN	sarcoma	PTEN loss, PDGFRA ampl	sorafenib	0.64	SD	4.46	5.72
04-0009	breast	UNKNOWN	AR, ESR1, PGR exp	abiraterone	357.46	PD	2.26	5.22
04-0010	UNKNOWN	adenocarcinoma	TP53-N239D; AR exp	abiraterone	348.14	SD	2.49	4.4
04-0012	breast	UNKNOWN	TP53-R248W; AR, ESR1, PGR exp; HER2 ampl	abiraterone	357.46	PD	0.46	16.72
04-0013	breast	UNKNOWN	KRAS-A146T, PIK3CA-E545K; ESR1, PGR exp; PTEN loss	everolimus	4165	PR	7.51	16.66
04-0019	ovary	adenocarcinoma	TP53-R273H; AR, ESR1 exp; STK11, INPP4B, PTEN loss	everolimus	3054.15	SD	5.64	17.94
04-0042	colon	adenocarcinoma	TP53-R196*, KRAS-G12delinsAG; PTEN, INPP4B loss	everolimus	176.09	SD	3.84	7.26
04-0047	ovary	adenocarcinoma	TP53-G279E; ESR1, AR exp; STK11, INPP4B loss	tamoxifen	2336.93	PD	1.8	4.93
05-0001	breast	UNKNOWN	APC-Q1447fs; AR exp; STK11, PTEN loss	abiraterone	357.46	PR	3.54	9.13
05-0005	breast	UNKNOWN	AR, ESR1 exp	abiraterone	357.46	PD	2.26	4.8
05-0013	breast	UNKNOWN	PIK3CA-E545K; AR, ESR1 exp	everolimus	4001.17	SD	5.18	20.99
05-0014	breast	UNKNOWN	PTEN-D324Y, TP53-S215fs; AR exp; STK11, INPP4B, PTEN loss	abiraterone	357.46	PD	1.64	11.63
05-0015	UNKNOWN	urothelial carcinoma	TP53-E285K; AR exp; PTEN, STK11, INPP4B loss; HER2 ampl	lapatinib + trastuzumab	3501.065	PD	2.07	8.41
05-0018	colon	adenocarcinoma	APC-E1353*, PIK3CA-E545K, TP53-R248Q, CSF1R-E317fs, SMAD4-450_451del; PTEN, STK11 loss, PDGFRB ampl	sorafenib	0.51	PD	2.3	19.81
05-0020	breast	UNKNOWN	TP53-Q165*; INPP4B, STK11 loss; RICTOR ampl, PTEN loss	everolimus	330.19	PD	1.8	14.36
05-0026	nasopharynx	UNKNOWN	AR exp	abiraterone	315.04	PD	1.67	10.18
05-0027	UNKNOWN	urothelial carcinoma	ESR1 exp; PTEN loss	tamoxifen	2284.13	PD	1.93	12.52
05-0028	breast	UNKNOWN	TP53-E204*; INPP4B, STK11, PTEN loss	everolimus	330.19	PD	2.85	22.11
05-0032	lung	UNKNOWN	TP53-P278R, FLT3-M665T; INPP4B, STK11 loss; RICTOR ampl	sorafenib	191.61	SD	5.93	13.37
05-0040	ovary	adenocarcinoma	AR, ESR1 exp; STK11, PTEN, INPP4B loss; PIK3CA ampl	everolimus	3054.47	SD	4.07	11.96
05-0046	cervix	squamous cell carcinoma	AKT1-E17K, KRAS-G12V, TP53-111_111del	everolimus	-642.39	PD	1.48	9.66
06-0011	breast	UNKNOWN	TP53-D207fs; PTEN loss	everolimus	313.66	PD	1.84	9.1
06-0014	breast	UNKNOWN	PIK3CA-H1047R, TP53-R248G	everolimus	1391.17	SD	3.8	11.5
06-0022	lung	UNKNOWN	KIT-D572G	imatinib	329.18	SD	8.36	14
06-0027	breast	UNKNOWN	PTEN, STK11, INPP4B loss, PIK3CA ampl	everolimus	435.54	PD	1.84	8.15
06-0041	ovary	adenocarcinoma	PIK3CA-E542K, TP53-M133K; STK11, INPP4B loss	everolimus	449.9	PD	1.57	10.38
06-0042	UNKNOWN	urothelial carcinoma	ABL1-K247R, FGFR3-S249C, AR exp	abiraterone	315.04	SD	6.43	7.85
06-0044	cervix	squamous cell carcinoma	FBXW7-R505C, PIK3CA-V346G; ESR1 exp	tamoxifen	2263.9	SD	3.51	15.51
06-0047	colon	adenocarcinoma	KRAS-G12V, TP53-R282W; INPP4B, PTEN loss	everolimus	-1291.37	PD	1.57	3.48
06-0052	nasopharynx	UNKNOWN	ESR1, PGR exp	tamoxifen	2284.13	SD	3.74	13.8
06-0056	cervix	squamous cell carcinoma	PIK3CA-E545K; ESR1, PGR exp	everolimus	498.45	PD	2.07	6.05
06-0063	colon	adenocarcinoma	BRAF-V600E, FBXW7-R465C	vemurafenib	21997.84	PR	5.57	11.27
06-0075	pancreas	adenocarcinoma	KRAS-G12D, TP53-R282W, CDKN2A-P81L; INPP4B, PTEN loss	everolimus	-996.6	PD	1.64	3.68
06-0079	colon	adenocarcinoma	KRAS-G12V, TP53-A159V, PTEN loss	everolimus	-1291.37	PD	2.03	5.03
06-0090	lung	UNKNOWN	TP53-R273L, SMO-F187V; ESR1 exp; SRC ampl; PTEN loss	everolimus	3001.97	PD	1.8	6.7
06-0094	ovary	adenocarcinoma	KIT-V852I	imatinib	329.03	SD	3.7	11.1
06-0096	endometrium	adenocarcinoma	ATM-V410A, KRAS-G12V, PIK3CA-E545K, PTEN-Y68H, PTEN-F337C, APC-1501_1505del; ESR1, PGR exp; PTEN, STK11, INPP4B loss	everolimus	1930.6	SD	5.93	8.34
06-0103	lung	UNKNOWN	EGFR-T790M, TP53-191_194del; PGR exp; INPP4B loss; EGFR ampl	tamoxifen	0.23	SD	3.41	18.33
06-0105	endometrium	adenocarcinoma	STK11 loss, PIK3CA ampl, PTEN loss	everolimus	379.06	SD	9.18	21.52
07-0006	UNKNOWN	adenoid cystic carcinoma	TP53-G245C; KIT, PDGFRA, RICTOR ampl	sorafenib	0.49	PD	4	12.16
07-0011	pancreas	adenocarcinoma	AR, PGR exp	abiraterone	348.14	SD	9.87	20.83
07-0014	breast	UNKNOWN	PIK3CA-N345K; ESR1, AR exp; STK11, INPP4B loss	abiraterone	357.46	PD	2.07	10.55
07-0037	endometrium	adenocarcinoma	KRAS-G12V, PIK3CA-E542K	everolimus	-949.51	SD	15.28	29.8
07-0044	UNKNOWN	germ cell tumors	STK11, PTEN loss	everolimus	265.98	SD	8.07	11.99
07-0046	UNKNOWN	sarcoma	AR, PGR exp; PTEN loss	everolimus	249.45	SD	5.7	9.89
07-0053	colon	adenocarcinoma	APC-R1450*, APC-R876*, JAK3-V722I, KRAS-G13D, PIK3CA-E545K, TP53-R282W; STK11, INPP4B loss; HER2 ampl	everolimus	-224.52	PD	2.43	9.17
07-0057	ovary	adenocarcinoma	ATM-R2719H; AR, ESR1 exp; INPP4B loss	tamoxifen	2336.93	SD	5.77	27.6
07-0070	ovary	adenocarcinoma	ESR1 exp	tamoxifen	2336.93	SD	18.23	24.9
07-0081	bone	osteosarcoma	AR exp	abiraterone	315.04	PD	2.16	26.09
07-0112	colon	adenocarcinoma	APC-1295_1296del; STK11, PTEN, INPP4B loss	everolimus	266.93	SD	3.61	8.74
07-0116	colon	adenocarcinoma	KRAS-G12D, PIK3CA-E545K, APC-1462_1463del	everolimus	-796.87	PD	1.51	7.29
07-0124	endometrium	adenocarcinoma	RET-E623K; ESR1 exp; PTEN loss	everolimus	3012.87	SD	5.74	21.85
07-0126	pancreas	adenocarcinoma	PTEN, INPP4B loss	everolimus	250.01	PD	3.48	7.1
07-0136	ovary	adenocarcinoma	CTNNB1-T41I, PIK3CA-H1047R; ESR1, PGR exp	everolimus	3572.15	SD	4.79	12.68
07-0141	eye	uveal melanoma	GNAQ-Q209P; AR exp; STK11 loss	abiraterone	315.04	PD	2.16	6.7
07-0154	gastroesophageal junction	adenocarcinoma	TP53-H179Y; PGR exp	tamoxifen	0.23	SD	18	20.01
08-0009	ovary	adenocarcinoma	APC-L1302fs, APC-R1450fs, AKT1-L156delinsLW, TP53-N345fs, TP53-T155fs; INPP4B, PTEN, STK11 loss; YES1, PIK3CA ampl	everolimus	382.87	PD	2.13	11.17
08-0018	cervix	squamous cell carcinoma	ESR1 exp; STK11 , PTEN loss; PIK3CB, PIK3CA ampl	tamoxifen	2178.56	PD	2.75	12.52
08-0020	ovary	adenocarcinoma	TP53-R196*; ESR1, PGR exp	tamoxifen	2336.93	SD	3.87	9.56
08-0024	endometrium	adenocarcinoma	AKT1-E17K, APC-R1450*, EGFR-E97K, PIK3CA-P458L; ESR1, PGR exp	everolimus	2792.87	PD	4.59	12.12

Supplementary Table 2. DDA of a case (Example 5) in more detail than in Table 1. Tables includes the DDA AEL scores of associated drivers to targets and associated drivers and targets.

patient ID	tumor type	DRIVER	AEL	TARGET	AEL	inherited driver items (AEL)			sum inherited AEL
Example 5	breast cancer	ER OVEREXPRESSION	731.820	PIK3CA WILD-TYPE	1251.37	ER OVEREXPRESSION (731.82)	PIK3CA-E545K (276.63)		1008.45
		PIK3CA-E545K	276.630	ER WILD-TYPE	1222.00	ER OVEREXPRESSION (731.82)	PIK3CA-E545K (-276.63)		455.19
		KRAS-A146T	150.910	mTOR WILD-TYPE	1102.61	ER OVEREXPRESSION (731.82)	PIK3CA-E545K (276.63)	PTEN LOSS (50.04)	1058.49
		PTEN LOSS	50.040	AKT1 WILD-TYPE	1013.79	ER OVEREXPRESSION (731.82)	PIK3CA-E545K (276.63)		1008.45
		PR OVEREXPRESSION	5.500	CDK4 WILD-TYPE	888.34	ER OVEREXPRESSION (731.82)	KRAS-A146T (150.92)		882.74
				CDK6 WILD-TYPE	883.44	ER OVEREXPRESSION (731.82)	KRAS-A146T (150.92)		882.74
				AKT2 WILD-TYPE	277.74	PIK3CA-E545K (276.63)			276.63
				CTNNB1 WILD-TYPE	277.07	PIK3CA-E545K (276.63)			276.63
				AKT3 WILD-TYPE	276.99	PIK3CA-E545K (276.63)			276.63
				RAF1 WILD-TYPE	154.78	KRAS-A146T (150.92)			150.92
				PLK1 WILD-TYPE	153.74	KRAS-A146T (150.92)			150.92
				SOS1 WILD-TYPE	152.75	KRAS-A146T (150.92)			150.92
				HSP90 WILD-TYPE	152.62	KRAS-A146T (150.92)			150.92
				MAPK1 WILD-TYPE	152.36	KRAS-A146T (150.92)			150.92
				MAPK3 WILD-TYPE	152.36	KRAS-A146T (150.92)			150.92
				CNKSR1 WILD-TYPE	151.76	KRAS-A146T (150.92)			150.92
				DNMT1 WILD-TYPE	151.76	KRAS-A146T (150.92)			150.92
				CDK1 WILD-TYPE	151.21	KRAS-A146T (150.92)			150.92
				PDL1 WILD-TYPE	50.52	PTEN LOSS (50.04)			50.04
				EGFR MUTANT	-50.73	PTEN LOSS (-50.04)			-50.04
				PARP1 WILD-TYPE	-101.56	KRAS-A146T (-150.92)	PTEN LOSS (50.04)		-100.88
				MAP2K1 WILD-TYPE	-115.93	KRAS-A146T (150.92)	PIK3CA-E545K (-276.63)		-125.71
				BRAF WILD-TYPE	-158.39	KRAS-A146T (-150.92)			-150.92
				ERBB2 WILD-TYPE	-334.38	PIK3CA-E545K (-276.63)	PTEN LOSS (-50.04)		-326.67
				EGFR WILD-TYPE	-675.79	KRAS-A146T (-150.92)	PIK3CA-E545K (-276.63)		-427.55

COMPOUND	AEL	inherited driver items (AEL)				sum inherited driver AEL	inherited target items (AEL)			sum inherited target AEL	sum inherited driver+target AEL
PALBOCICLIB	4604.32	ER OVEREXPRESSION (731.82)	KRAS-A146T (150.92)			882.74	CDK4 WILD-TYPE (888.34)	CDK6 WILD-TYPE (883.44)		1771.78	2654.52
LETROZOLE	3898.00	ER OVEREXPRESSION (731.82)				731.82	ER WILD-TYPE (1222.00)			1222.00	1953.82
ABEMACICLIB	3080.47	ER OVEREXPRESSION (731.82)	KRAS-A146T (150.92)			882.74	CDK4 WILD-TYPE (888.34)	CDK6 WILD-TYPE (883.44)		1771.78	2654.52
RIBOCICLIB	3058.48	ER OVEREXPRESSION (731.82)				731.82	CDK4 WILD-TYPE (888.34)	CDK6 WILD-TYPE (883.44)		1771.78	2503.6
EVEROLIMUS	2886.78	ER OVEREXPRESSION (731.82)	KRAS-A146T (150.92)	PIK3CA-E545K (276.63)	PTEN LOSS (50.04)	907.57	mTOR WILD-TYPE (1102.61)			1102.61	2010.18
TAMOXIFEN	2773.34	ER OVEREXPRESSION (731.82)				731.82	ER WILD-TYPE (1222.00)			1222.00	1958.82
GSK2126458	2634.61	PIK3CA-E545K (276.63)				276.63	mTOR WILD-TYPE (1102.61)	PIK3CA WILD-TYPE (1251.37)		2353.98	2630.61
DACTOLISIB	2632.83	PIK3CA-E545K (276.63)				276.63	mTOR WILD-TYPE (1102.61)	PIK3CA WILD-TYPE (1251.37)		2353.98	2630.61
FULVESTRANT	2472.13	ER OVEREXPRESSION (731.82)				731.82	ER WILD-TYPE (1222.00)			1222.00	1958.82
VOXTALISIB	2354.17						mTOR WILD-TYPE (1102.61)	PIK3CA WILD-TYPE (1251.37)		2353.98	2353.98
PWT33597	2353.98						mTOR WILD-TYPE (1102.61)	PIK3CA WILD-TYPE (1251.37)		2353.98	2353.98
PI-103	2353.98						mTOR WILD-TYPE (1102.61)	PIK3CA WILD-TYPE (1251.37)		2353.98	2353.98
VS-5584	2353.98						mTOR WILD-TYPE (1102.61)	PIK3CA WILD-TYPE (1251.37)		2353.98	2353.98
PKI179	2353.98						mTOR WILD-TYPE (1102.61)	PIK3CA WILD-TYPE (1251.37)		2353.98	2353.98
SF1126	2353.98						mTOR WILD-TYPE (1102.61)	PIK3CA WILD-TYPE (1251.37)		2353.98	2353.98
GEDATOLISIB	2353.98						mTOR WILD-TYPE (1102.61)	PIK3CA WILD-TYPE (1251.37)		2353.98	2353.98
PF-04691502	2353.98						mTOR WILD-TYPE (1102.61)	PIK3CA WILD-TYPE (1251.37)		2353.98	2353.98
P7170	2353.98						mTOR WILD-TYPE (1102.61)	PIK3CA WILD-TYPE (1251.37)		2353.98	2353.98
APITOLISIB	2353.98						mTOR WILD-TYPE (1102.61)	PIK3CA WILD-TYPE (1251.37)		2353.98	2353.98
DS-7423	2353.98						mTOR WILD-TYPE (1102.61)	PIK3CA WILD-TYPE (1251.37)		2353.98	2353.98
BGT226	2353.98						mTOR WILD-TYPE (1102.61)	PIK3CA WILD-TYPE (1251.37)		2353.98	2353.98
TASELISIB	2287.02	ER OVEREXPRESSION (731.82)	PIK3CA-E545K (276.63)			1008.45	PIK3CA WILD-TYPE (1251.37)			1251.37	2259.82
ALPELISIB	2268.62	ER OVEREXPRESSION (731.82)	PIK3CA-E545K (276.63)			1008.45	PIK3CA WILD-TYPE (1251.37)			1251.37	2259.82
AZD9496	2040.32	ER OVEREXPRESSION (731.82)				731.82	ER WILD-TYPE (1222.00)			1222.00	1958.82
GDC-0810	2008.22	ER OVEREXPRESSION (731.82)				731.82	ER WILD-TYPE (1222.00)			1222.00	1958.82
ELACESTRANT	1997.07	ER OVEREXPRESSION (731.82)				731.82	ER WILD-TYPE (1222.00)			1222.00	1958.82
EXEMESTANE	1984.30	ER OVEREXPRESSION (731.82)				731.82	ER WILD-TYPE (1222.00)			1222.00	1958.82
RONICICLIB	1922.99						CDK4 WILD-TYPE (888.34)	CDK6 WILD-TYPE (883.44)	CDK1 WILD-TYPE (151.21)	1922.99	1922.100
IPATASERTIB	1569.74						AKT1 WILD-TYPE (1013.79)	AKT2 WILD-TYPE (277.74)	AKT3 WILD-TYPE (276.99)	1568.52	1568.53
GDC-0077	1538.00	PIK3CA-E545K (276.63)				276.63	PIK3CA WILD-TYPE (1251.37)			1251.37	276.63
PAXALISIB	1531.18	PIK3CA-E545K (276.63)				276.63	PIK3CA WILD-TYPE (1251.37)			1251.37	276.63
PICTILISIB	1529.93	PIK3CA-E545K (276.63)				276.63	PIK3CA WILD-TYPE (1251.37)			1251.37	276.63
BUPARLISIB	1529.02	PIK3CA-E545K (276.63)				276.63	PIK3CA WILD-TYPE (1251.37)			1251.37	276.63
XL147	1528.95	PIK3CA-E545K (276.63)				276.63	PIK3CA WILD-TYPE (1251.37)			1251.37	276.63
COPANLISIB	1528.55	PIK3CA-E545K (276.63)				276.63	PIK3CA WILD-TYPE (1251.37)			1251.37	276.63
TEMSIROLIMUS	1447.09	PIK3CA-E545K (276.63)				326.67	mTOR WILD-TYPE (1102.61)			1102.61	326.67
SIROLIMUS	1388.14	PIK3CA-E545K (276.63)				276.63	mTOR WILD-TYPE (1102.61)			1102.61	276.63
METFORMIN	1380.59	PIK3CA-E545K (276.63)				276.63	mTOR WILD-TYPE (1102.61)			1102.61	276.63
CH 5132799	1251.37						PIK3CA WILD-TYPE (1251.37)			1251.37	1251.37
AZD6482	1251.37						PIK3CA WILD-TYPE (1251.37)			1251.37	1251.37
RECILISIB	1251.37						PIK3CA WILD-TYPE (1251.37)			1251.37	1251.37
WX 037	1251.37						PIK3CA WILD-TYPE (1251.37)			1251.37	1251.37
SONOLISIB	1251.37						PIK3CA WILD-TYPE (1251.37)			1251.37	1251.37
MLN1117	1251.37						PIK3CA WILD-TYPE (1251.37)			1251.37	1251.37
ZSTK474	1251.37						PIK3CA WILD-TYPE (1251.37)			1251.37	1251.37
GSK1059615	1251.37						PIK3CA WILD-TYPE (1251.37)			1251.37	1251.37
BAZEDOXIFENE	1222.21						ER WILD-TYPE (1222.00)			1222.00	1222.00
GDC-0927	1222.00						ER WILD-TYPE (1222.00)			1222.00	1222.00
RIDAFOROLIMUS	1103.28						mTOR WILD-TYPE (1102.61)			1102.61	1102.61
VISUSERTIB	1102.61						mTOR WILD-TYPE (1102.61)			1102.61	1102.61
RES 529	1102.61						mTOR WILD-TYPE (1102.61)			1102.61	1102.61
GDC 0349	1102.61						mTOR WILD-TYPE (1102.61)			1102.61	1102.61
PANULISIB	1102.61						mTOR WILD-TYPE (1102.61)			1102.61	1102.61
CC-115	1102.61						mTOR WILD-TYPE (1102.61)			1102.61	1102.61
MLN0128	1102.61						mTOR WILD-TYPE (1102.61)			1102.61	1102.61
AZD8055	1102.61						mTOR WILD-TYPE (1102.61)			1102.61	1102.61
OSI-027	1102.61						mTOR WILD-TYPE (1102.61)			1102.61	1102.61
CC-223	1102.61						mTOR WILD-TYPE (1102.61)			1102.61	1102.61
AZD2014	1102.61						mTOR WILD-TYPE (1102.61)			1102.61	1102.61
RIVICICLIB	1039.55						CDK4 WILD-TYPE (888.34)	CDK1 WILD-TYPE (151.21)		1039.55	1039.55
RGB-286638	1039.55						CDK4 WILD-TYPE (888.34)	CDK1 WILD-TYPE (151.21)		1039.55	1039.55
ALVOCIDIB	1039.55						CDK4 WILD-TYPE (888.34)	CDK1 WILD-TYPE (151.21)		1039.55	1039.55
MILCICLIB	1039.55						CDK4 WILD-TYPE (888.34)	CDK1 WILD-TYPE (151.21)		1039.55	1039.55
MK2206	1013.97						AKT1 WILD-TYPE (1013.79)			1013.79	1013.79
DANUSERTIB	1013.79						AKT1 WILD-TYPE (1013.79)			1013.79	1013.79
SR13668	1013.79						AKT1 WILD-TYPE (1013.79)			1013.79	1013.79
GSK2141795	1013.79						AKT1 WILD-TYPE (1013.79)			1013.79	1013.79
AFURESERTIB	1013.79						AKT1 WILD-TYPE (1013.79)			1013.79	1013.79
BAY1125976	1013.79						AKT1 WILD-TYPE (1013.79)			1013.79	1013.79
CAPIVASERTIB	1013.79						AKT1 WILD-TYPE (1013.79)			1013.79	1013.79
AT13148	1013.79						AKT1 WILD-TYPE (1013.79)			1013.79	1013.79
ARQ 092	1013.79						AKT1 WILD-TYPE (1013.79)			1013.79	1013.79
SC-66	1013.79						AKT1 WILD-TYPE (1013.79)			1013.79	1013.79
TRICIRIBINE	1013.79						AKT1 WILD-TYPE (1013.79)			1013.79	1013.79
GSK690693	1013.79						AKT1 WILD-TYPE (1013.79)			1013.79	1013.79
ASPIRIN	356.39	PIK3CA-E545K (276.63)				276.63					276.63
GANETESPIB	305.58	KRAS-A146T (150.92)				150.92	HSP90 WILD-TYPE (152.63)			152.63	303.55
BAY-293	304.56	KRAS-A146T (150.92)				150.92	SOS1 WILD-TYPE (152.76)			152.76	303.68
E7386	277.07						CTNNB1 WILD-TYPE (277.07)			277.07	277.07
PRI-724	277.07						CTNNB1 WILD-TYPE (277.07)			277.07	277.07
ATEZOLIZUMAB	215.50	KRAS-A146T (150.92)				150.92	PDL1 WILD-TYPE (50.52)			50.52	201.44
NIVOLUMAB	168.34	KRAS-A146T (150.92)				150.92					150.92
PEMBROLIZUMAB	165.00	KRAS-A146T (150.92)				150.92					150.92
NEXIRI	157.95	KRAS-A146T (150.92)				150.92					150.92
ISIS 5132	154.78						RAF1 WILD-TYPE (154.78)			154.78	154.78
PLX 5568	154.78						RAF1 WILD-TYPE (154.78)			154.78	154.78
AT13387	154.36						HSP90 WILD-TYPE (152.63)			152.63	152.63
TAK-960	153.74						PLK1 WILD-TYPE (153.75)			153.75	153.75
NMS-P937	153.74						PLK1 WILD-TYPE (153.75)			153.75	153.75
GSK-461364	153.74						PLK1 WILD-TYPE (153.75)			153.75	153.75
BI 2536	153.74						PLK1 WILD-TYPE (153.75)			153.75	153.75
VOLASERTIB	153.74						PLK1 WILD-TYPE (153.75)			153.75	153.75
ONALESPIB	152.62						HSP90 WILD-TYPE (152.63)			152.63	152.63
17-AAG	152.62						HSP90 WILD-TYPE (152.63)			152.63	152.63
XL888	152.62						HSP90 WILD-TYPE (152.63)			152.63	152.63
SNX-5422	152.62						HSP90 WILD-TYPE (152.63)			152.63	152.63
MPC-3100	152.62						HSP90 WILD-TYPE (152.63)			152.63	152.63
KW-2478	152.62						HSP90 WILD-TYPE (152.63)			152.63	152.63
IPI-493	152.62						HSP90 WILD-TYPE (152.63)			152.63	152.63
HSP990	152.62						HSP90 WILD-TYPE (152.63)			152.63	152.63
DEBIO 932	152.62						HSP90 WILD-TYPE (152.63)			152.63	152.63
TANESPIMYCIN	152.62						HSP90 WILD-TYPE (152.63)			152.63	152.63
BIIB028	152.62						HSP90 WILD-TYPE (152.63)			152.63	152.63
BIIB021	152.62						HSP90 WILD-TYPE (152.63)			152.63	152.63
AUY922	152.62						HSP90 WILD-TYPE (152.63)			152.63	152.63
ABI-010	152.62						HSP90 WILD-TYPE (152.63)			152.63	152.63
GDC 0994	152.36						MAPK1 WILD-TYPE (152.37)			152.37	152.37
MK 8353	152.36						MAPK1 WILD-TYPE (152.37)			152.37	152.37
BMS-690514	151.79	KRAS-A146T (150.92)				150.92					150.92
AMG 510	151.51	KRAS-A146T (150.92)				150.92					150.92
CISPLATIN	151.26	KRAS-A146T (150.92)				150.92					150.92
AT 7519	151.21						CDK1 WILD-TYPE (151.21)			151.21	151.21
TG02	151.21						CDK1 WILD-TYPE (151.21)			151.21	151.21
DINACICLIB	151.21						CDK1 WILD-TYPE (151.21)			151.21	151.21
AZD5438	151.21						CDK1 WILD-TYPE (151.21)			151.21	151.21
SORAFENIB	148.05	KRAS-A146T (150.92)				150.92	RAF1 WILD-TYPE (154.78)	BRAF WILD-TYPE (-154.80)		-3.62	147.3
SELUMETINIB	55.27	KRAS-A146T (150.92)				150.92	MAP2K1 WILD-TYPE (-115.93)			-115.93	34.99
AVELUMAB	50.72						PDL1 WILD-TYPE (50.52)			50.52	50.52
MDX-1105	50.52						PDL1 WILD-TYPE (50.52)			50.52	50.52
PD0325901	42.48	KRAS-A146T (150.92)				150.92	MAP2K1 WILD-TYPE (-115.93)			-115.93	34.99
PIMASERTIB	40.70	KRAS-A146T (150.92)				150.92	MAP2K1 WILD-TYPE (-115.93)			-115.93	34.100
TRAMETINIB	39.27	KRAS-A146T (150.92)				150.92	MAP2K1 WILD-TYPE (-115.93)			-115.93	34.101
BINIMETINIB	35.50	KRAS-A146T (150.92)				150.92	MAP2K1 WILD-TYPE (-115.93)			-115.93	34.102
BEVACIZUMAB	12.99										
BGB-283	9.92	KRAS-A146T (150.92)				150.92	BRAF WILD-TYPE (-158.40)			-158.40	7.48
AXICABTAGENE CILOLEUCEL	9.01										
PAZOPANIB	4.52										
TAS-102	4.08										
DECITABINE	3.38										
ENZALUTAMIDE	2.30										
RAMUCIRUMAB	2.26										
ZIV-AFLIBERCEPT	2.13										
OBINUTUZUMAB	2.13										
BORTEZOMIB	2.09										
LENVATINIB	2.04										
DINUTUXIMAB	2.02										
ABIRATERONE	1.98										
NAB-PACLITAXEL	1.98										
FOLFIRINOX	1.94										
CEDIRANIB	1.64										
DENOSUMAB	1.43										
IPILIMUMAB	1.13										
TREBANANIB	1.10										
NINTEDANIB	1.08										
VENETOCLAX	0.98										
BELANTAMAB MAFODOTIN	0.90										
ORTERONEL	0.90										
ENFORTUMAB VEDOTIN-EJFV	0.67										
APALUTAMIDE	0.51										
OLARATUMAB	0.49										
IXAZOMIB	0.43										
RUXOLITINIB	0.41										
CABAZITAXEL	0.36										
SONIDEGIB	0.32										
NICLOSAMIDE	0.29										
VORINOSTAT	0.29										
DAROLUTAMIDE	0.28										
SILMITASERTIB	0.21										
AICAR	0.21										
AXITINIB	0.20										
SACITUZUMAB GOVITECAN	0.20										
DINUTUXIMAB BETA	0.20										
RIPRETINIB	0.20										
AVAPRITINIB	0.20										
CARBOPLATIN	0.20										
TOFACITINIB	0.19										
ETOPOSIDE	0.18										
NUTLIN-3A	0.16										
RILUZOLE	0.16										
CRIZOTINIB	0.15										
LUCITANIB	0.14										
FLUVASTATIN	0.12										
ATORVASTATIN	0.12										
OCTREOTIDE	0.12										
PENTOSTATIN	0.12										
TRICHOSTATIN A	0.12										
ENTINOSTAT	0.12										
MEDI-573	0.10										
ENTRECTINIB	0.10										
HER2-BBz-CAR T cells	0.10										
LCAR-B38M	0.10										
DOSTARLIMAB	0.10										
CABOZANTINIB	0.10										
ZOLEDRONIC ACID	0.07										
AZD4547	0.07										
NILOTINIB	0.06										
PROPRANOLOL	0.05										
MIDOSTAURIN	0.05										
LOBENGUANE	0.01										
DASATINIB	0.00										
HDM201	0.00										
CCT3833	-2.68						RAF1 WILD-TYPE (154.78)	BRAF WILD-TYPE (-158.40)		-3.62	-3.62
REGORAFENIB	-2.78						RAF1 WILD-TYPE (154.78)	BRAF WILD-TYPE (-158.40)		-3.62	-3.62
LXH254	-3.62						RAF1 WILD-TYPE (154.78)	BRAF WILD-TYPE (-158.40)		-3.62	-3.62
HM95573	-3.62						RAF1 WILD-TYPE (154.78)	BRAF WILD-TYPE (-158.40)		-3.62	-3.62
LY-3009120	-3.62						RAF1 WILD-TYPE (154.78)	BRAF WILD-TYPE (-158.40)		-3.62	-3.62
TAK 580	-3.62						RAF1 WILD-TYPE (154.78)	BRAF WILD-TYPE (-158.40)		-3.62	-3.62
XL281	-3.62						RAF1 WILD-TYPE (154.78)	BRAF WILD-TYPE (-158.40)		-3.62	-3.62
ARQ 736	-3.62						RAF1 WILD-TYPE (154.78)	BRAF WILD-TYPE (-158.40)		-3.62	-3.62
VELIPARIB	-21.52	PTEN LOSS (50.04)				50.04	PARP1 WILD-TYPE (-101.56)			-101.56	-51.52
BRIGATINIB	-50.73						EGFR MUTANT (-50.73)			-50.73	-50.73
NIRAPARIB	-96.58						PARP1 WILD-TYPE (-101.56)			-101.56	-101.56
FOLFOX	-100.64	KRAS-A146T (150.92)	PIK3CA-E545K (-276.63)			-125.71					-125.71
RUCAPARIB	-100.86						PARP1 WILD-TYPE (-101.56)			-101.56	-101.56
2X-121	-101.56						PARP1 WILD-TYPE (-101.56)			-101.56	-101.56
AZD 2461	-101.56						PARP1 WILD-TYPE (-101.56)			-101.56	-101.56
INO-1001	-101.56						PARP1 WILD-TYPE (-101.56)			-101.56	-101.56
FLUZOPARIB	-101.56						PARP1 WILD-TYPE (-101.56)			-101.56	-101.56
PAMIPARIB	-101.56						PARP1 WILD-TYPE (-101.56)			-101.56	-101.56
E7449	-101.56						PARP1 WILD-TYPE (-101.56)			-101.56	-101.56
E7016	-101.56						PARP1 WILD-TYPE (-101.56)			-101.56	-101.56
CEP-9722	-101.56						PARP1 WILD-TYPE (-101.56)			-101.56	-101.56
INIPARIB	-101.56						PARP1 WILD-TYPE (-101.56)			-101.56	-101.56
TALAZOPARIB	-101.56						PARP1 WILD-TYPE (-101.56)			-101.56	-101.56
ABT767	-101.56						PARP1 WILD-TYPE (-101.56)			-101.56	-101.56
OLAPARIB	-101.94						PARP1 WILD-TYPE (-101.56)			-101.56	-101.56
COBIMETINIB	-115.87						MAP2K1 WILD-TYPE (-115.93)			-115.93	-115.93
BI-847325	-115.93						MAP2K1 WILD-TYPE (-115.93)			-115.93	-115.93
PD98059	-115.93						MAP2K1 WILD-TYPE (-115.93)			-115.93	-115.93
ARRY-300	-115.93						MAP2K1 WILD-TYPE (-115.93)			-115.93	-115.93
AS703988	-115.93						MAP2K1 WILD-TYPE (-115.93)			-115.93	-115.93
U0126	-115.93						MAP2K1 WILD-TYPE (-115.93)			-115.93	-115.93
WX-554	-115.93						MAP2K1 WILD-TYPE (-115.93)			-115.93	-115.93
TAK-733	-115.93						MAP2K1 WILD-TYPE (-115.93)			-115.93	-115.93
RO4987655	-115.93						MAP2K1 WILD-TYPE (-115.93)			-115.93	-115.93
GDC-0623	-115.93						MAP2K1 WILD-TYPE (-115.93)			-115.93	-115.93
E6201	-115.93						MAP2K1 WILD-TYPE (-115.93)			-115.93	-115.93
REFAMETINIB	-115.93						MAP2K1 WILD-TYPE (-115.93)			-115.93	-115.93
AZD8330	-115.93						MAP2K1 WILD-TYPE (-115.93)			-115.93	-115.93
CI-1040	-119.52						MAP2K1 WILD-TYPE (-115.93)			-115.93	-115.93
RO5126766	-119.55						MAP2K1 WILD-TYPE (-115.93)	RAF1 WILD-TYPE (154.78)	BRAF WILD-TYPE (-158.40)	-119.55	-119.55
IMATINIB	-150.01	KRAS-A146T (-150.92)				-150.92					-150.92
SUNITINIB	-151.04	KRAS-A146T (-150.92)				-150.92					-150.92
PEMETREXED	-151.24	KRAS-A146T (-150.92)				-150.92					-150.92
SALIRASIB	-153.58	KRAS-A146T (-150.92)				-150.92					-150.92
GEMCITABINE	-154.71	KRAS-A146T (-150.92)				-150.92					-150.92
ENCORAFENIB	-158.39						BRAF WILD-TYPE (-158.40)			-158.40	-158.40
DOXORUBICIN	-276.82	PIK3CA-E545K (-276.63)				-276.63					-276.63
LAPATINIB	-310.27	PIK3CA-E545K (-276.63)				-276.63	AKT1 WILD-TYPE (1013.79)	ERBB2 WILD-TYPE (-334.38)	EGFR WILD-TYPE (-675.79)	3.62	-273.01
DABRAFENIB	-311.45	KRAS-A146T (-150.92)				-150.92	BRAF WILD-TYPE (-158.40)			-158.40	-309.32
VEMURAFENIB	-311.61	KRAS-A146T (-150.92)				-150.92	BRAF WILD-TYPE (-158.40)			-158.40	-309.32
SYD985	-334.11						ERBB2 WILD-TYPE (-334.38)			-334.38	-334.38
TRASTUZUMAB EMTANSINE	-334.19						ERBB2 WILD-TYPE (-334.38)			-334.38	-334.38
MM-302	-334.21						ERBB2 WILD-TYPE (-334.38)			-334.38	-334.38
POZIOTINIB	-334.38						ERBB2 WILD-TYPE (-334.38)			-334.38	-334.38
TRASTUZUMAB DERUXTECAN	-334.38						ERBB2 WILD-TYPE (-334.38)			-334.38	-334.38
VARLITINIB	-334.38						ERBB2 WILD-TYPE (-334.38)			-334.38	-334.38
MDX-210	-334.38						ERBB2 WILD-TYPE (-334.38)			-334.38	-334.38
PYROTINIB	-334.38						ERBB2 WILD-TYPE (-334.38)			-334.38	-334.38
MUBRITINIB	-334.38						ERBB2 WILD-TYPE (-334.38)			-334.38	-334.38
PERTUZUMAB	-334.38						ERBB2 WILD-TYPE (-334.38)			-334.38	-334.38
MARGETUXIMAB	-334.38						ERBB2 WILD-TYPE (-334.38)			-334.38	-334.38
CP-724714	-334.38						ERBB2 WILD-TYPE (-334.38)			-334.38	-334.38
CANERTINIB	-334.38						ERBB2 WILD-TYPE (-334.38)			-334.38	-334.38
BMS-599626	-334.38						ERBB2 WILD-TYPE (-334.38)			-334.38	-334.38
TUCATINIB	-334.38						ERBB2 WILD-TYPE (-334.38)			-334.38	-334.38
VANDETANIB	-674.87						EGFR WILD-TYPE (-675.79)			-675.79	-675.79
NECITUMUMAB	-675.38						EGFR WILD-TYPE (-675.79)			-675.79	-675.79
ZALUTUMUMAB	-675.71						EGFR WILD-TYPE (-675.79)			-675.79	-675.79
SIMOTINIB	-675.79						EGFR WILD-TYPE (-675.79)			-675.79	-675.79
MATUZUMAB	-675.79						EGFR WILD-TYPE (-675.79)			-675.79	-675.79
H 447	-675.79						EGFR WILD-TYPE (-675.79)			-675.79	-675.79
IMGATUZUMAB	-675.79						EGFR WILD-TYPE (-675.79)			-675.79	-675.79
BIBX 1382	-675.79						EGFR WILD-TYPE (-675.79)			-675.79	-675.79
PKI 166	-675.79						EGFR WILD-TYPE (-675.79)			-675.79	-675.79
THELIATINIB	-675.79						EGFR WILD-TYPE (-675.79)			-675.79	-675.79
TESEVATINIB	-675.79						EGFR WILD-TYPE (-675.79)			-675.79	-675.79
JNJ-26483327	-675.79						EGFR WILD-TYPE (-675.79)			-675.79	-675.79
NIMOTUZUMAB	-675.79						EGFR WILD-TYPE (-675.79)			-675.79	-675.79
MEHD7945A	-675.79						EGFR WILD-TYPE (-675.79)			-675.79	-675.79
OSIMERTINIB	-675.79						EGFR WILD-TYPE (-675.79)			-675.79	-675.79
SAPITINIB	-675.79						EGFR WILD-TYPE (-675.79)			-675.79	-675.79
AEE788	-675.79						EGFR WILD-TYPE (-675.79)			-675.79	-675.79
TRASTUZUMAB	-738.57	PTEN LOSS (-50.04)	PIK3CA-E545K (-276.63)			-326.67	ERBB2 WILD-TYPE (-334.38)			-334.38	-661.05
GEFITINIB	-841.29	KRAS-A146T (-150.92)				-150.92	EGFR WILD-TYPE (-675.79)			-675.79	-826.71
NERATINIB	-1010.01						ERBB2 WILD-TYPE (-334.38)	EGFR WILD-TYPE (-675.79)		-1010.17	-1010.17
EPERTINIB	-1010.17						ERBB2 WILD-TYPE (-334.38)	EGFR WILD-TYPE (-675.79)		-1010.17	-1010.17
TAK-285	-1010.17						ERBB2 WILD-TYPE (-334.38)	EGFR WILD-TYPE (-675.79)		-1010.17	-1010.17
PELITINIB	-1010.17						ERBB2 WILD-TYPE (-334.38)	EGFR WILD-TYPE (-675.79)		-1010.17	-1010.17
CUDC-101	-1010.17						ERBB2 WILD-TYPE (-334.38)	EGFR WILD-TYPE (-675.79)		-1010.17	-1010.17
AV-412	-1010.17						ERBB2 WILD-TYPE (-334.38)	EGFR WILD-TYPE (-675.79)		-1010.17	-1010.17
ALLITINIB	-1010.17						ERBB2 WILD-TYPE (-334.38)	EGFR WILD-TYPE (-675.79)		-1010.17	-1010.17
AFATINIB	-1060.72						ERBB2 WILD-TYPE (-334.38)	EGFR WILD-TYPE (-675.79)	EGFR MUTANT (-50.73)	-1060.90	-1060.90
CETUXIMAB	-1110.83	KRAS-A146T (-150.92)	PIK3CA-E545K (-276.63)			-427.55	EGFR WILD-TYPE (-675.79)			-675.79	-1103.34
ERLOTINIB	-1130.73	KRAS-A146T (-150.92)	PIK3CA-E545K (-276.63)			-427.55	EGFR WILD-TYPE (-675.79)			-675.79	-1103.34
DACOMITINIB	-1215.03	KRAS-A146T (-150.92)				-150.92	ERBB2 WILD-TYPE (-334.38)	EGFR WILD-TYPE (-675.79)	EGFR MUTANT (-50.73)	-1060.90	-1211.82
PANITUMUMAB	-1487.93	KRAS-A146T (-150.92)	PIK3CA-E545K (-276.63)			-427.55	EGFR WILD-TYPE (-675.79)			-675.79	-1103.34

SUPPLEMENTARY FIGURE LEGEND

Supplementary Figure 1. Distribution histogram of AEL values of MTAs. The cases were grouped in intervals of 250 AELs to identify groups of patients with similar AELs.

SUPPLEMENTARY FIGURE

Supplementary Figure 1.

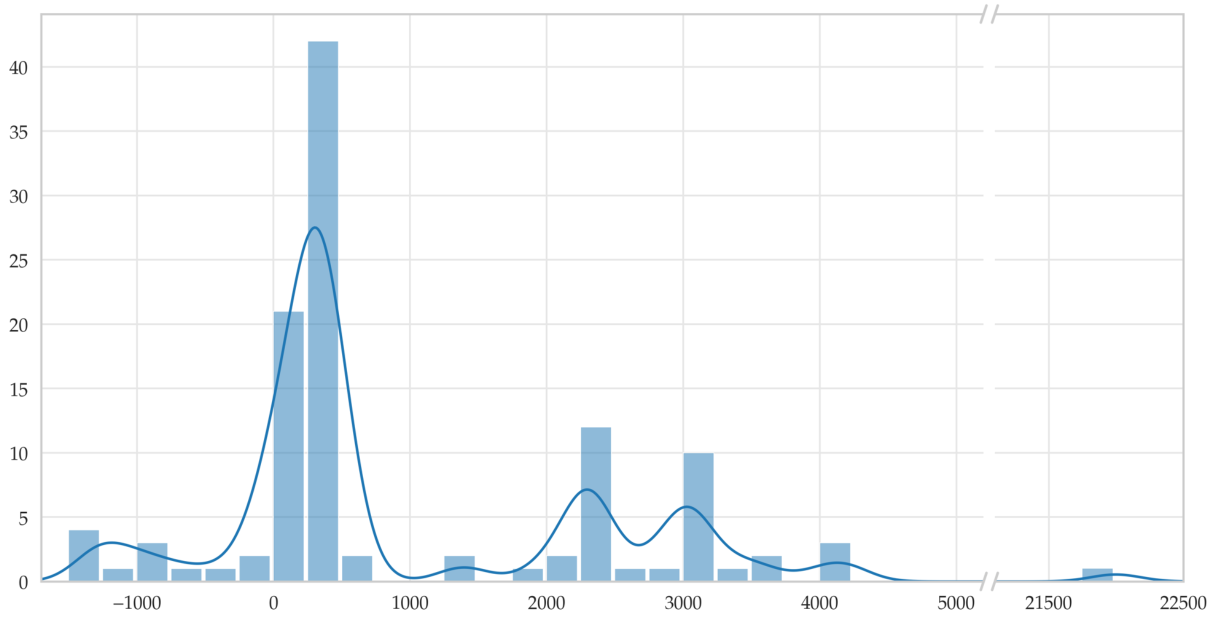

SUPPLEMENTARY NOTE 1

EXAMPLES FOR THE CLINICAL USE OF DDA

The user of the system registers the case in the case management system of the DDA software system. Next, uploads the results of next generating sequencing (NGS) sequencing panels (both hot spot focus panels and commercially available comprehensive panels) to the system manually from the test's report, or if the variant list (vcf) is available in excel format, the whole variant list automatically. Results of copy number variations generated by NGS and fluorescent in situ hybridization (FISH) (For example, HER-2), gene expression data by immunohistochemistry (IHC) (for example, PDL-1), single-gene sequencing (Sanger, Real-Time

PCR) are also added. MSI status and TMB (Tumor Mutational Burden) can also be added to the molecular profile. Next, the treatment calculator module of the DDA is started. The system automatically calculates an AEL score of each genetic alterations, related target, and targeted therapy and creates a prioritized list of treatment options based on the whole available molecular profile.

The system can help to make a decision between registered treatment options in the next line therapy of the specific tumor type or between off-label therapies related to the molecular profile.

Example 1.
A 50 years old woman was diagnosed with neuroendocrine pancreas cancer with liver metastasis. The clinican's question was which therapy to choose for the first line, and later after registered treatment options fail. NGS analysis revealed the following profile: PIK3CA-P539R, TP53-C135F. MUTYH-Y179C TSC2-E532*, TSC2-P542R, MLH1-K617N. The DDA identified 78 MTAs positively associated with this profile. The top MTAs based on the assigned AEL values were alpelisib, copanlisib (PI3K inhibitors), everolimus, bevacizumab, metformin, sunitinib. Based on these results, everolimus was used for first-line therapy as the drug with the highest AEL value out of the registered MTAs in this tumor type. The patient had a dramatic response to everolimus. In case, of progression, on label sunitinib or off-label treatment options targeting the PI3K are available. This is an example for quick, objective decision support in clinical setting between registered treatment options (everolimus, sunitinib and chemotherapy).

Example 2.
A 78 years old woman with metastatic lung adenocarcinoma was diagnosed. IHC of PDL-1 was negative; the NGS analysis revealed low TMB, FGFR1, and CDK4 amplification and NRG-1-translocation, among other alterations. DDA identified FGFR amplification as the most important driver, the most important target, and nintedanib as the MTA with the highest AEL registered on this tumor type targeting FGFR. The next most important driver was calculated to be the NRG-1 (neuregulin) translocation. The most important target related to NRG-1 is ERBB3/HER-3 receptor, according to the DDA. DDA assigned afatinib to target ERBB3.

Interestingly, nivolumab and other checkpoint inhibitors registered in lung adenocarcinomas received negative AEL values due to the negative association between the NRG-1 translocation and PD1/PDL-1 inhibitors. Based on these results of DDA, the patient's personalized treatment plan was to receive chemotherapy and bevacizumab as first-line therapy without immune checkpoint inhibitor; for the second line, the plan is the registered combination of nintedanib-docetaxel in lung cancer, the third line is afatinib off label therapy. This example shows that DDA can make objective decisions between targeted therapies and targeted therapies and immune checkpoint inhibitors.

Example 3.

A 40-year-old lady was diagnosed with metastatic breast cancer. The tumor was triple negative (ERBB2/HER-2 FISH negative, ER, PR IHC negative). Subsequently, it was analyzed with a comprehensive NGS panel. The results indicated a point mutation in the tyrosine kinase domain of the ERBB2 gene, ERBB-2-G776V, BRCA2-T3033_FS, MYC, and RAD21 amplification. The question of the molecular tumor board was whether to target the ERBB-2 alteration with an HER-2 inhibitor or to use PARP inhibitor first. The DDA ranked the alterations based on the calculated AEL score: BRCA2-T3033_FS, MYC, ERBB-2-G776V, RAD21. The ranking order of the related targets was ERBB-2, PDL-1, PARP, BRD4, CDK1, BET, CHECK, CDK12. The ranking order of the MTAs (the top out of 63 possible MTAs) was olaparib, TDM-1, trastuzumab, neratinib, talazoparib, and PDL-1 inhibitors in combination with PARP inhibitors in clinical trials. The system classified the BRCA as the most significant driver alteration and identified the ERBB2 as the most important target in breast cancer, but still selected the PARP inhibitor related to BRCA first, most probably because of the sensitivity of mutant ERBB2 shows significant variability to different ERBB2 inhibitors. This case represents an example of the complexity of the clinical decision, even in the case of common driver alterations. In these cases, one by one drug assignment does not help to make the clinical decision.
