# Supplementary figures and images for: A computational method for prioritizing targeted therapies in precision oncology: performance analysis in the SHIVA01 trial
Source: NPJ Precis Oncol. 2021 Jun 23;5:59. doi: 10.1038/s41698-021-00191-2 (PMC8222375; doi:10.1038/s41698-021-00191-2)

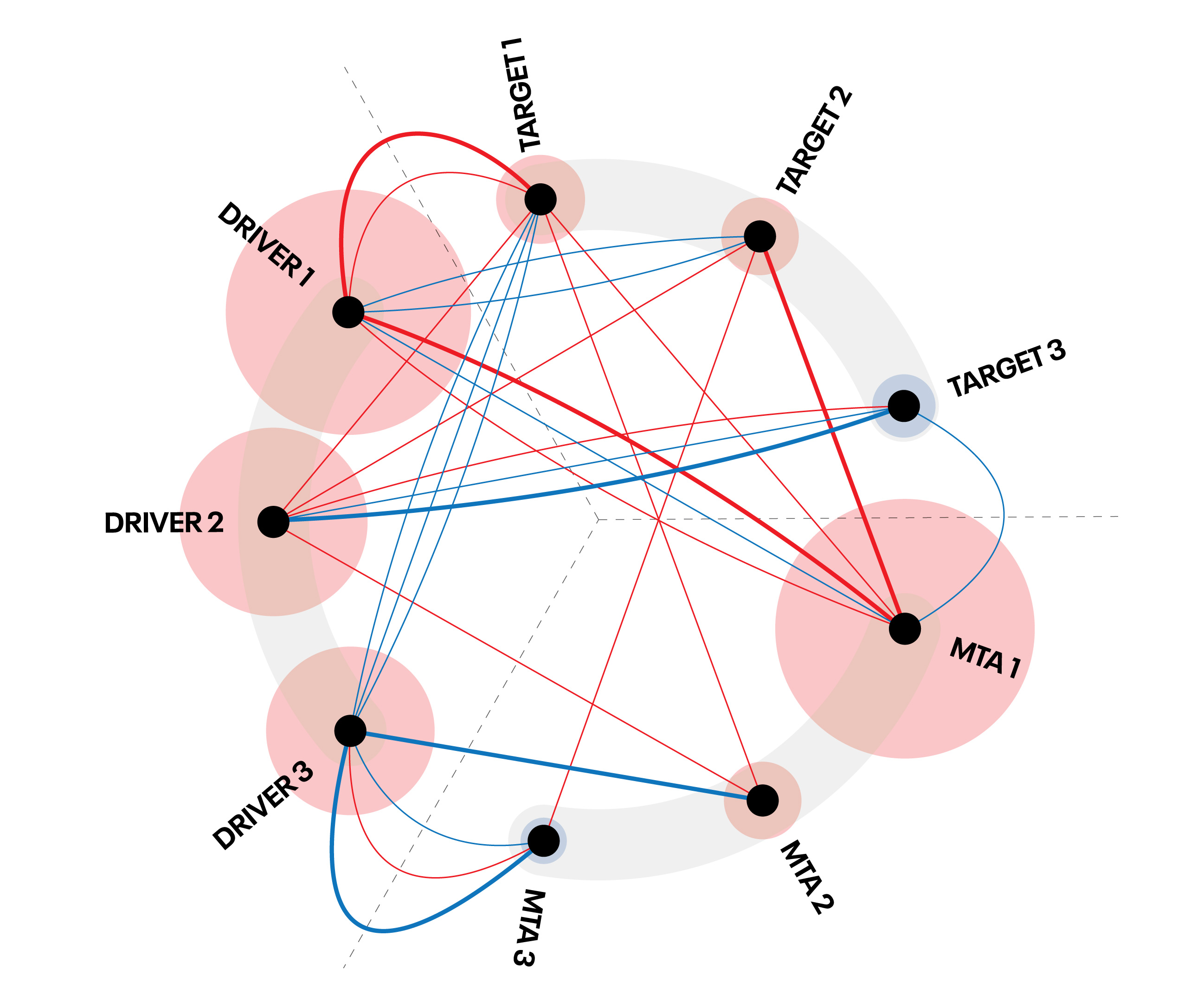

Supplement: Supplementary file 2 — Supplementary Data 1 [file 41698_2021_191_MOESM2_ESM.jpg]
